# METTL3‐stabilized super enhancers‐lncRNA SUCLG2‐AS1 mediates the formation of a long‐range chromatin loop between enhancers and promoters of SOX2 in metastasis and radiosensitivity of nasopharyngeal carcinoma

**DOI:** 10.1002/ctm2.1361

**Published:** 2023-09-01

**Authors:** Xinyu Hu, Jianfeng Wu, Yong Feng, Hongxia Ma, Erbao Zhang, Chang Zhang, Qi Sun, Tingting Wang, Yizhi Ge, Dan Zong, Wei Chen, Xia He

**Affiliations:** ^1^ Department of Radiotherapy The Afliated Cancer Hospital of Nanjing Medical University and Jiangsu Cancer Hospital and Jiangsu Institute of Cancer Research Nanjing China; ^2^ Department of Epidemiology and Biostatistics International Joint Research Center On Environment and Human Health, Center for Global Health, School of Public Health, Nanjing Medical University Nanjing China

**Keywords:** nasopharyngeal carcinoma, radiosensitivity, SOX2, SUCLG2‐AS1, super‐enhancer

## Abstract

**Background:**

Super enhancers (SE) play pivotal roles in cell identity and diseases occur including tumorigenesis. The depletion of SE‐associated lncRNA transcripts, also known as super‐lncRNA, causes the activity of SE to be dysregulated.

**Methods:**

We screened and identified an elevated metastasis‐associated SE‐lncRNA SUCLG2‐AS1 in nasopharyngeal carcinoma (NPC) using RNA‐sequencing, real‐time quantitative polymerase chain reaction (RT‐qPCR) and bioinformatics. Western blotting, RT‐qPCR, methylated RNA immunoprecipitation (MeRIP), RNA immunoprecipitation, chromatin immunoprecipitation, RNA pull‐down and 3C (chromosome conformation capture assays) were used for mechanistic studies.

**Results:**

SUCLG2‐AS1 was correlated with a poor prognosis. SUCLG2‐AS1 promotes NPC cell invasion and metastasis while repressing apoptosis and radiosensitivity in vitro and in vivo. Mechanistically, high SUCLG2‐AS1 expression occurred in an m6A‐dependent manner. SUCLG2‐AS1 was found to be located in the SE region of SOX2, and it regulated the expression of SOX2 via long‐range chromatin loop formation, which via mediating CTCF (transcription factor) occupied the SE and promoter region of SOX2, thus regulating the metastasis and radiosensitivity of NPC.

**Conclusions:**

Taken together, our data suggest that SUCLG2‐AS1 may serve as a novel intervention target for the clinical treatment of NPC.

## Graphical abstract

- SUCLG2‐AS1 was upregulated in nasopharyngeal carcinoma (NPC) tissues and associated with poor prognosis in NPC patients.
- SUCLG2‐AS1 promotes cell invasion and metastasis while repressing apoptosis and radiosensitivity in vitro and in vivo.
- The writing of SUCLG2‐AS1 m6A modified promotes RNA stability of SUCLG2‐AS1.
- Upregulated SUCLG2‐AS1 activates expression of SOX2 via long‐range chromatin loop formation, which via mediating CTCF occupied super‐enhancer and promoter region of SOX2 by SUCLG2‐AS1.

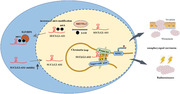

## 1 INTRODUCTION

Nasopharyngeal carcinoma (NPC) is common in southern China but rare in other regions, with 80 000 incident cases and 50 000 deaths occurring annually.^1^, ^2^ Distant metastasis is the main crux of treatment failure and, is currently a major clinical challenge.^3^ In addition to this, radioresistance has been a critical problem limiting the efficacy of NPC treatment and leading to recurrence.^4^ Therefore, it is necessary to explore the molecular mechanisms underlying the metastasis and treatment of NPC as well as potential markers or intervention targets.

Recent studies indicated that lncRNA expression is frequently dysregulated in many cancers.^5^ Although lncRNA is not translated into protein, it has been well established that lncRNA regulates coding gene expression through several mechanisms, including transcriptional and post‐transcriptional processing.^6^ Recently, super‐enhancer‐associated lncRNAs (SE‐lncRNAs) have been identified. There are specific types of lncRNAs that are transcribed from SE or interact with SE.^7^ SE‐lncRNAs can regulate gene expression through long‐range chromatin interactions or the chromatin loop.^8^ Dysregulated SE‐lncRNA expression can be involved in multiple biological processes including tumorigenesis.^9^, ^10^ For instance, the expression of LINC00339 was regulated by long‐range chromatin loop formation, which is involved in bone metabolism.^11^ SE‐lncRNA CCAT1‐L interacted with CTCF and mediated the formation of enhancer loops to activate MYC expression, which participates in the malignant progression of colorectal cancer.^8^ Nevertheless, the role of SE‐lncRNAs in NPC metastasis and radioresistance has not yet been elucidated.

Through transcriptome sequencing and integrated data analysis of NPC with and without metastatic lymph nodes, we screened and identified an elevated SE‐lncRNA SUCLG2‐AS1. There are few studies on this SE‐lncRNA. Thus, the purpose of our study was to establish the function and molecular mechanism of SUCLG2‐AS1 in NPC metastasis and development.^12^ We found that SUCLG2‐AS1 regulated metastasis and radiosensitivity in vitro and in vivo. METTL3‐mediated m6A modification occurs in SUCLG2‐AS1 transcripts, followed by the recognition and stabilization of IGF2BP3. SUCLG2‐AS1 regulates the expression of SOX2 via long‐range chromatin loop formation, thereby regulating NPC metastasis and radiosensitivity. Taken together, our results indicate that SUCLG2‐AS1 may serve as a novel target in NPC clinical treatment.

## 2 MATERIALS AND METHODS

### 2.1 NPC cell lines and clinical tissue samples

The NPC cell lines CNE1, 6−10B, CNE2 and 5−8F, as well as the human immortalized normal nasopharyngeal epithelial cell line (NP69) were purchased from ATCC. All cells were cultured in RPMI‐1640 medium containing 10% fetal bovine serum (BioChannel, China) and maintained at 37°C in 5% CO_2_ in an incubator. Sixty specimens of fresh pathologically confirmed NPC tissues and 10 cases of adjacent cancer were obtained from the Jiangsu Cancer Hospital. This study was subscribed to by the Ethics Committee of Jiangsu Cancer Hospital.

### 2.2 Transcriptome sequencing

We collected two pairs of cancer and paracancerous tissues from NPC patients for microarray detection of non‐coding RNA. After the total RNA in the tissue was extracted, rRNA was removed using a deribosome kit, and the remaining RNA was fragmented (approximately 200 nt) and reverse‐transcribed to synthesize single‐stranded complementary DNA (cDNA). Then double‐stranded cDNA was synthesized and purified. The ends were repaired, primers were supplied, and PCR amplification and purification were performed. Subsequently, the library was subjected to quality inspection, and finally sequenced. For the detected lncRNAs, lncRNA expression levels were calculated and the differentially expressed lncRNAs between samples were analyzed.

## 3 5′ AND 3′ RACE ASSAY

We used 5′ and 3′ RACE (rapid amplification of cDNA ends) kits (Invitrogen) to confirm the full‐length following of SUCLG2‐AS1 by the manufacturer's instructions. SUCLG2‐AS1 specific primers for RACE are displayed in the Supporting Information.

### 3.1 Transfection with plasmids and siRNAs

Three ASOs targeting SUCLG2‐AS1 and matched control ASONC were obtained from RuiboBi. SUCLG2‐AS1, SUCLG2‐AS1 mutant, CTCF plasmid, and pcDNA3.1 vector were constructed using Genecreat (Wuhan). Plasmids were transfected with X‐tremeGENE (Roche) and siRNA was transfected using Lipofectamine imax (Invitrogen) according to the manufacturer's instructions.

### 3.2 RNA extraction and cDNA synthesis

Total RNA from cells or tissues was isolated using the Trizol Reagent (Life Technologies). Then, cDNA was generated utilizing the PrimeScript RT Master mix (TaKaRa). Subsequently, quantitative RT‐PCR was executed using Green Master Mix (Invitrogen) and examined using a real‐time PCR instrument (ABI 7500 Fast; ABI) with GAPDH as the internal control. Relative RNA expression levels (fold changes) were reckoned using the 2^−△△Ct^ method. The primer sequences used are described in the Supporting Information.

### 3.3 Western blot analysis

Proteins were collected from the cells using RIPA lysis buffer mixed with a 1% Protease Inhibitor Cocktail. Antibodies against SOX2 (Proteintech, 1:1000), CTCF (CST, 1:1000), METTL3 (Proteintech,1:1000), MTEEL14 (Proteintech,1:1000), KIAA1429 (ABclonal, 1:1000), WTAP (Santa Claus, 1:500), FTO(Santa Claus,1:500), ALKBH5 (Santa Claus, 1:500), IGF2BP1 (Santa Claus, 1:500), IGF2BP2 (Santa Claus, 1:500), IGF2BP3 (Santa Claus, 1:500), HNRNPA2B1 (Proteintech, 1:1000), HUR (Santa Claus, 1:500),γ‐H2AX ( CST, 1:1000), cleaved Caspase‐3 (Abcam, 1:1000), Caspase‐3(CST, 1:1000), survivin (Santa Claus, 1:500) were used. After 30–60 min, the PVDF membranes were incubated with secondary antibodies and then, visualized using the ECL system (Tanon, China).

### 3.4 Subcellular localization analysis

A PARIS kit (Invitrogen) was utilized to insulate the nuclear and cytoplasmic RNA. GAPDH and U6 served as negative controls for cytoplasmic and nuclear RNA, respectively.

### 3.5 RNA fluorescence in situ hybridization

The labelled SUCLG2‐AS1 fluorescent probe was compounded by RuiboBio using a kit (RuiboBio) according to the manufacturer's instructions.

### 3.6 Migration, invasion, cell proliferation and colony formation assays

Functional experiments were conducted as described in our previous article.^13^

### 3.7 Radiosensitivity assay

Single‐cell suspensions were subcultured in six‐well plates. A 6 MeV electron beam produced by a linear accelerator was used for irradiation at a dose rate of 400 cGy/min. The distance between the source and the skin was 100 cm. Cells were subjected to gradient fraction doses of 2, 4 or 6 Gy using a variable collimator. After irradiation with different doses of γ‐rays, the cells were cultivated for about 14 days until colonies appeared (colonies are defined as > 50 cells) and the surviving colonies were counted. The cell survival curve was drawn using the single‐hit multitarget model: y = (1−e^−D/D0^)^n^ using GraphPad Prism 11 software. The radiation dose‐response curve was constructed by fitting data to the linear quadratic equation S = e^−αD‐βD^2^.

### 3.8 Cell apoptosis assay

The Annexin V‐FITC Apoptosis Detection kit (Beyotime) was used to quantify the apoptotic rate of CNE1 cells using flow cytometry (BD Biosciences) with BD CellQuest software.

### 3.9 RNA stability assay

Cells were treated with actinomycin D (MCE, 5 μg/ml) for 0, 3 and 6 h before RNA extraction and qRT‐PCR detection.

### 3.10 Methylated RNA immunoprecipitation

The level of m6A modification in SUCLG2‐AS1 and SUCLG2‐AS1 mutants was examined using a Magna methylated RNA immunoprecipitation (MeRIP) m6A kit (Millipore) following the manufacturer's instructions. An anti‐m6A antibody (Abcam) was used for the MeRIP assays. The enrichment of m6A was analyzed using qRT‐PCR.

### 3.11 RNA‐binding immunoprecipitation assay

An EZ‐Magna RNA immunoprecipitation (RIP) assay kit (Millipore) was used to perform the RIP assay. The magnetic beads were aggregated, resuspended and washed with the RIP wash buffer. Anti‐CTCF (CST; 7 μg), anti‐IGF2BP3 (CST; 7 μg) or anti‐IgG (Millipore; 5 μg) antibodies were used for the assay. The RNA bound to the magnetic beads was examined by real‐time quantitative polymerase chain reaction (RT‐qPCR).

### 3.12 RNA pull‐down assay and mass spectrometry analysis

The sense and antisense sequences of SUCLG2‐AS1 were synthetized in vitro using the MEGAscript T7 Transcription kit (Thermo Fisher Scientific). SUCLG2‐AS1 and antisense SUCLG2‐AS1 were transcribed in vitro from pcDNA3.1‐SUCLG2‐AS1 using T7 RNA polymerase and purified using the AxyPrep PCR Clean‐up kit (Axygen). The transcribed RNA was labelled with biotin utilizing a Pierce RNA 3′ End Desthiobiotinylation kit (Thermo Fisher Scientific). Finally, the eluted proteins were examined by western blot analysis.

### 3.13 Luciferase promoter assay

NPC cells were co‐transfected with the pGL3‐SOX2 promoter, pGL3‐promoter‐E1, pGL3‐promoter‐E2, pGL3‐promoter‐E3, pGL3‐promoter‐E4, or pGL3‐promoter‐E1+2+3+4, which were constructed using the luciferase reporter vector pGL3‐promoter (Promega). pGL3‐vector was used as the internal control. Luciferase reporter assay was performed using a Dual‐Luciferase Reporter Assay System kit (Promega, E1960). The ratio of *Renilla* luciferase activity to firefly luciferase activity was used for normalization. And luciferase signal was measured using Spectra Max M5e (Molecular Devices).

### 3.14 Chromatin Immunoprecipitation

Chromatin Immunoprecipitation (ChIP) assays were executed by the EZ‐Magna Chip A/G Chromatin Immunoprecipitation kit (Millipore) according to the manufacturer's protocols. Antibodies targeting CTCF, SOX2 and IgG were used in each assay. Free DNA was eluted. Purified DNA was subjected to qRT‐PCR.

### 3.15 Chromosome conformation capture

The 3C protocol has been previously described by Sun et al.^14^ *Dpn* II was used to digest the genomic DNA. The ligation products were quantified by touchdown PCR using sequence‐specific primers (A: 5′‐ATACAAGGTCCATTCCCCCG‐3′, B: 5′‐TGGACTTCTTTTTGGGGGACTAT‐3′, C:5′‐ACACCAAACAGGTGTGGGAA‐3′, and D: 5′‐CACAATCGC TGGTCCTGTGT‐3′).

### 3.16 Mice xenograft and tumour metastasis assay

Four to six weeks old female BALB/c‐nu mice were cultured under specific pathogen‐free conditions. CNE1 cells (2×10^6^) transfected with shSUCLG2‐AS1, or negative control were suspended in 20 μl saline and implanted subcutaneously into the mice. After four weeks, the subcutaneous tumour reached approximately 50 mm^3^ and the mice were randomly assigned to four groups (n = 6/group): negative control, shSUCLG2‐AS1, negative control + IR (10 Gy), and shSUCLG2‐AS1 + IR (10 Gy). Each mouse was remedied with a total dose of 10 Gy at a dose rate of 3.62 Gy/min. Tumour volumes were calculated every three days using the following equation: (length×width^2^)/2.

For the lymph node metastasis model, CNE1 cells (5×10^6^) transfected with shSUCLG2‐AS1 or control were suspended in 20 μl saline and injected into the foot pads of nude mice. On the 30th day, the mice were euthanized, and their tumours and axillary lymph nodes were dissected.

### 3.17 Statistical analysis

The data from the research are presented as the mean ± SD. Comparisons between two groups were analyzed by a two‐sided unpaired t‐test or one‐way analysis of variance. The correlation between shSUCLG2‐AS1 and control groups in the lymph node metastasis model was analyzed using the chi‐squared test. Overall survival (OS) and progression‐free survival (PFS) were analyzed using the Kaplan‐Meier method. A *P*‐value lower than 0.05 and 0.001 indicated statistical significance.

## 4 RESULTS

### 4.1 Screening and identification of metastasis‐associated super‐lncRNA in NPC

**FIGURE 1:**
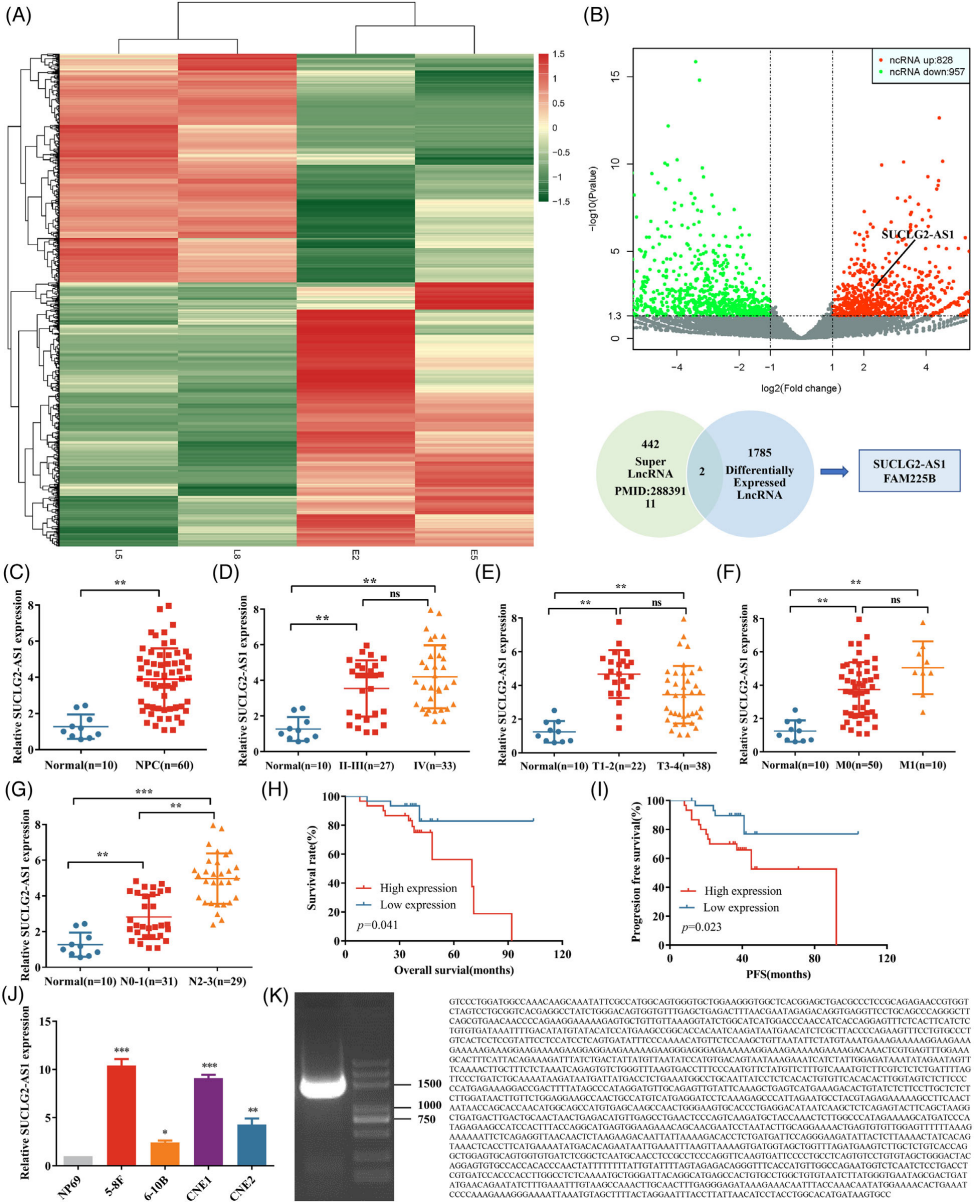
Screening and identification of metastasis‐associated SE‐lncRNA in nasopharyngeal carcinoma (NPC). (**A** and **B**) Heat map and Volcano map of NPC tissue samples with differentially expressed genes. L5 and L8 represent NPC tissues of the N_2‐3_ stage. E2 and E5 represent NPC tissues of the N_0‐1_ stage. (**C**) qRT‐PCR assays were used to test the expression levels of SUCLG2‐AS1 in 10 normal nasopharyngeal and 60 NPC tissue samples. (**D–G**) The expression of SUCLG2‐AS1 was assayed in normal nasopharyngeal tissues and tumour tissues at different stages. (**H**) Kaplan‐Meier curve analysis of the impact of SUCLG2‐AS1 expression on overall survival (OS) of patients with NPC. (**I**) The Kaplan‐Meier curve analysis of the impact of SUCLG2‐AS1 expression on progression‐free survival (PFS) of patients with NPC. (**J**) The expression level of SUCLG2‐AS1 in NP69 and NPC cell lines was determined by qRT‐PCR. (**K**) The full sequence of SUCLG2‐AS1 was confirmed by RACE. *p < 0.05 and **p < 0.01. ns: not significant.

In this study, we performed genome‐wide lncRNA differential expression profile screening in two pairs of fresh tissue samples from NPC patients with metastatic lymph nodes and primary tumours using a microarray to identify novel and promising differentially expressed lncRNAs in NPC. A total of 1785 lncRNAs in metastatic tumour tissues showed a significant difference in expression (more than 2 times), of which 828 were highly expressed and 957 were expressed at low levels (Figure 1A,B). Recently, a new type of lncRNA transcribed from or interacting with SE —super‐lncRNA, has been recognized. In 2017, an American research team identified 442 SE lncRNA transcripts in 27 human cells and tissues.^12^ We used the intersection of 442 super‐lncRNA (https://rnajournal.cshlp.org/content/23/11/1729.full) and 1785 differentially expressed lncRNAs for data analysis, and two super lncRNAs (SUCLG2‐AS1 and FAM225B) were obtained. Compared with its expression in 10 normal tissues, SUCLG2‐AS1 was significantly upregulated in 60 NPC tumor tissues different stages (Figure 1C–F). However, the expression of FAM225B in clinical samples was inhomogeneous (data not shown). SUCLG2‐AS1 has recently attracted our attention. SUCLG2‐AS1 expression levels were notably positively correlated with the N stage of NPC (Figure 1G). We also analyzed the correlation between SUCLG2‐AS1 expression and OS and PFS of NPC (Figure 1H,I). The higher the expression, the shorter the patient's survival duration and the worse the prognosis. The primary clinicopathological characteristics of the NPC cases are summarized in Tables 1, 2, 3. In addition, compared with its expression in NP69, SUCLG2‐AS1 expression was elevated in NPC cell lines, especially in the 5−8F and CNE1 cell lines (Figure 1J). These results suggest that the SE‐lncRNA SUCLG2‐AS1 plays a role in the malignant progression and metastasis of NPC. The full‐length sequence of SUCLG2‐AS1 was amplified and confirmed by RACE experiment, and it was found that the total length of SUCLG2‐AS1 was 1984 bp (Figure 1K) with limited protein‐coding potential (https://lncipedia.org/; Figure S1A–C).

**TABLE 1:** Correlation between SUCLG2‐AS1 expression and clinical characteristics of nasopharyngeal carcinoma (NPC) patients.

Characteristics	No of patients (*n* = 60)	SUCLG2‐AS1 High group	SUCLG2‐AS1 Low group	*p*‐Value
Age (years)				
≤51	32	16	16	0.000
>51	28	14	14	
Gender				
female	17	5	12	0.046
male	43	25	18	
TNM stage				
II‐III	27	15	12	0.445
IV	33	15	18	
T classification				
T1‐T2	22	16	6	0.007
T3‐T4	38	14	24	
N classification				
N0‐N1	31	10	21	0.009
N2‐N3	29	20	9	
M classification				
M0	50	23	27	0.171
M1	10	7	3	

**TABLE 2:** Prognostic factors for overall survival (OS) and progression‐free survival (PFS) from univariate analysis.

Characteristics	No 60.	OS		PFS	
		HR (95%CI)	*p*	HR (95%CI)	*p*
Age(years)					
≤51	32	1		1	
>51	28	2.221 (0.719–6.860)	0.166	1.799 (0.640–5.060)	0.266
Gender					
Female	17	1		1	
Male	43	0.849 (0.257–2.812)	0.789	0.523 (0.182–1.498)	0.227
TMN stage					
II‐III	27	1		1	
IV	33	1.541 (0.500–4.744)	0.451	2.725 (0.865–8.588)	0.087
T classification					
T1‐2	22	1		1	
T3‐4	38	0.556 (0.183–1.686)	0.300	0.493 (0.178–1.368)	0.174
N classification					
N0‐1	31	1		1	
N2‐3	29	5.204 (1.144–23.681)	0.033	4.529 (1.277–16.066)	0.019
M classification					
M0	50	1		1	
M1	10	3.313 (1.018–10.781)	0.047	3.163 (1.039–9.632)	0.043
SUCLG2‐AS1 expression					
Low	30	1		1	
High	30	0.206 (0.045–0.936)	0.041	0.229 (0.065–0.815)	0.023

**TABLE 3:** Prognostic factors for overall survival (OS) and progression‐free survival (PFS) from multivariate analysis.

Characteristics	No 60.	OS		PFS	
		HR (95%CI)	*p*	HR (95%CI)	*p*
Age(years)					
≤51	32	1		1	
>51	28	2.946 (0.486–17.861)	0.240	1.772 (0.430–7.305)	0.429
Gender					
Female	17	1		1	
Male	43	0.225 (0.036–1.421)	0.113	0.258 (0.061–1.086)	0.065
TMN stage					
II‐III	27	1		1	
IV	33	0.677 (0.097–4.704)	0.693	3.018 (0.526–17.311)	0.215
T stage					
T1‐2	22	1		1	
T3‐4	38	1.047 (0.192–5.704)	0.956	0.608 (0.141–2.622)	0.505
N stage					
N0‐1	31	1		1	
N2‐3	29	5.763 (0.537–61.854)	0.148	2.116 (0.375–11.932)	0.396
M stage					
M0	50	1		1	
M1	10	1.876 (0.283–12.432)	0.514	0.727 (0.139–3.807)	0.706
SUCLG2‐AS1 expression					
Low	30	1		1	
High	30	0.185 (0.020–1.722)	0.138	0.172 (0.330–0.886)	0.035

### 4.2 Super‐lncRNA SUCLG2‐AS1 regulates cell migration and invasion of NPC in vitro and in vivo

**FIGURE 2:**
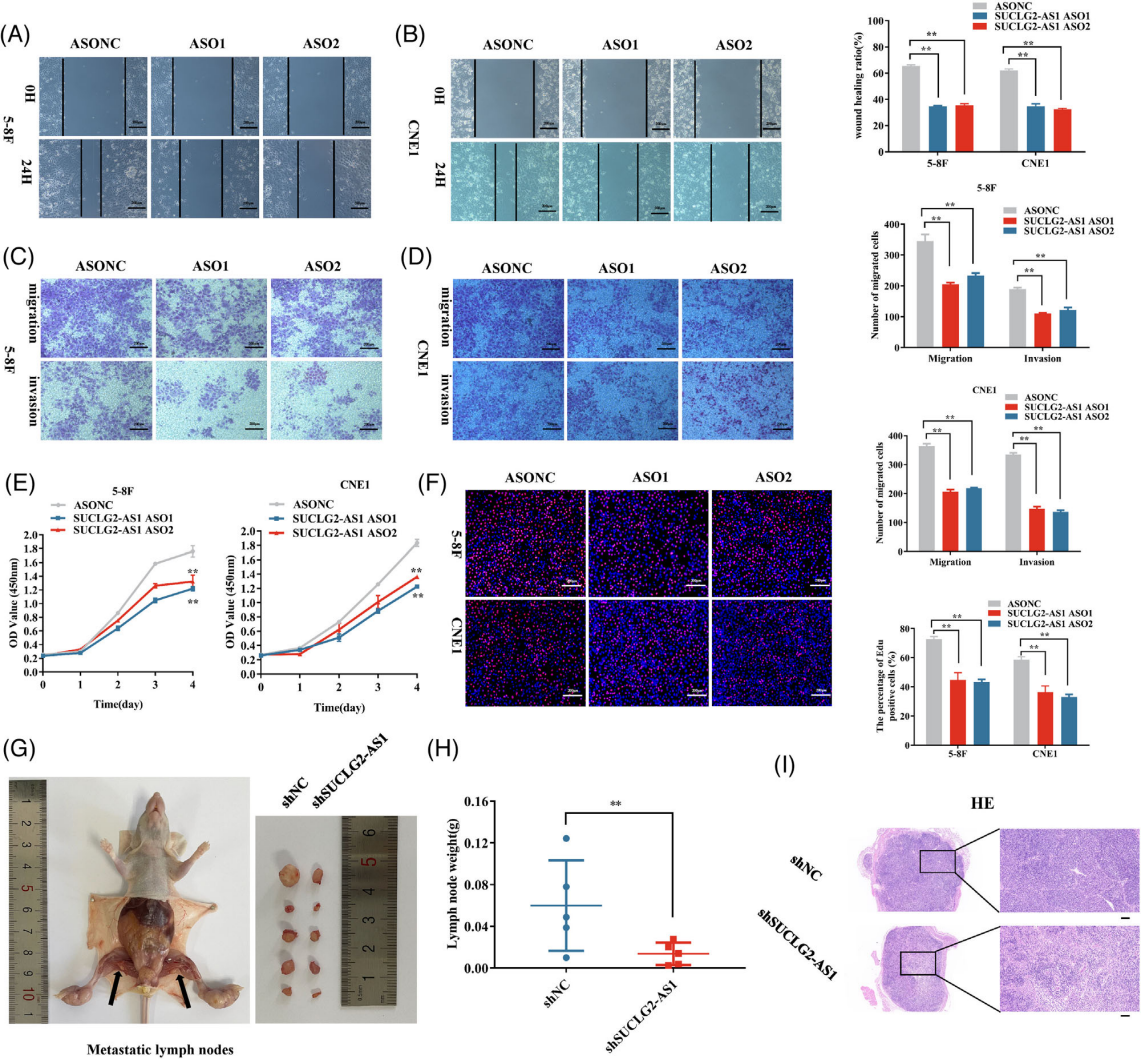
SE‐lncRNA SUCLG2‐AS1 regulates cell migration and invasion in vitro and in vivo in nasopharyngeal carcinoma (NPC). (**A–D**) Wound healing and transwell assay indicated that SUCLG2‐AS1 reduced the NPC cell ability to invade and migrate. (**E** and **F**) CCK8 and EDU assays were used to explore the cell viability of NPC cells. (**G**) Representative images of the metastatic popliteal lymph nodes in two groups. (**H**) The data indicated that shSUCLG2‐AS1 had significant effect on the average metastatic popliteal lymph nodes weight. (**I**) Metastatic nodules were subjected to H&E staining. Scale bar = 100 μm. The results presented are from three independent experiments. ASONC, ASO1 and ASO2 represent Antisense Oligonucleotides specific to SUCLG2‐AS1. *p < 0.05 and **p < 0.01.

To evaluate the biological role of SUCLG2‐AS1 in NPC, SUCLG2‐AS1 overexpression plasmid and antisense oligonucleotides (ASOs) were established in the 5−8F and CNE1 cell lines (Figure S2A,B). Silencing SUCLG2‐AS1 expression retarded invasion and migration in CNE1 and 5−8F cells compared to the internal control (Figure 2A–D). SUCLG2‐AS1 downregulation suppressed NPC cell proliferation (Figure 2E,F). To further examine the functions of SUCLG2‐AS1 in vivo, we constructed a xenograft model with stable lentivirus‐infected 5−8F cells. In the popliteal lymph node metastasis model, metastatic loci were obviously diminished in the shSUCLG2‐AS1 group compared with those in the shNC group (Figure 2G,H). Hematoxylin‐eosin (H&E) staining and IHC with anti‐pan‐cytokeratin showed that the metastatic lymph nodes loci were indeed tumour tissue (Figure 2I and Figure S2D). Altogether, these results demonstrate that SUCLG2‐AS1 influences proliferation, invasion and metastasis in vitro and in vivo by functioning as a tumour metastasis‐promoting lncRNA.

### 4.3 METTL3‐mediated m6A modification stabilizes SUCLG2‐AS1 expression in an IGF2BP3‐dependent manner

**FIGURE 3:**
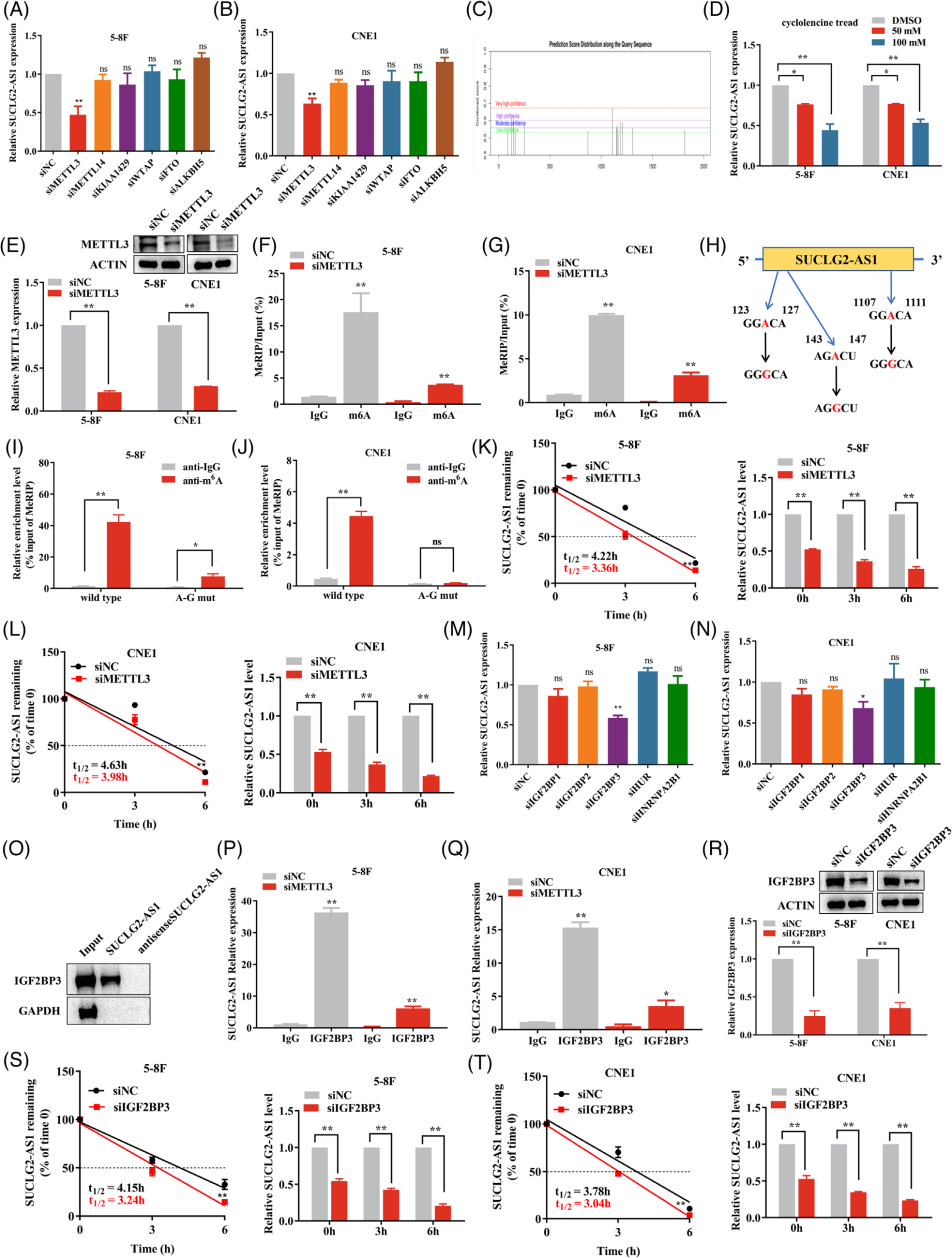
METTL3‐mediated m6A modification stabilizes SUCLG2‐AS1 expression in an IGF2BP3‐dependent manner. (**A** and **B**) The expression of SUCLG2‐AS1 after the knockdown of common m6A writers was examined. (**C**) SRAMP predicted the m6A modification site on SUCLG2‐AS1. (**D**) qRT‐PCR assays were used to examine cycloleucine‐treated 5−8F and CNE1 cells. (**E**) The knockdown efficiency of METTL3 was detected. (**F** and **G**) MeRIP‐qPCR assay was performed to analyze the m6A modification level of SUCLG2‐AS1 in nasopharyngeal carcinoma (NPC) cells after METTL3 knockdown. (**H**) SUCLG2‐AS1 m6A mutant (A–G) plasmid. (**I** and **J**) MeRIP‐qPCR assay was performed to analyze the m6A modification level on the transcript of SUCLG2‐AS1 mutant and wild type. (**K** and **L**) SUCLG2‐AS1 half‐life in METTL3‐silenced NPC cells. (**M** and **N**) The expression of SUCLG2‐AS1 after the knockdown of common m6A readers was detected. (**O**) RNA pull‐down assay was performed to analyze the specific binding of SUCLG2‐AS1 to IGF2BP3. (**P** and **Q**) RIP‐PCR was performed to analyze the expression of SUCLG2‐AS1 pulldown by IGF2BP3 antibody after knockdown of METTL3. (**R**) qRT‐PCR and western blot were used to detect the knockdown efficiency of IGF2BP3. (**S** and **T**) SUCLG2‐AS1 half‐life in IGF2BP3‐silenced NPC cells. *p < 0.05 and **p < 0.01.

Recent advances have demonstrated the critical role of m6A modifications in RNA expression and tumour progression.^15^ We observed the expression of SUCLG2‐AS1 after the knockdown of common m6A writers and erasers (METTL3, METTL14, KIAA1429, WTAP, FTO, and ALKBH5). Knockdown efficiencies were successfully determined (Figure S3A,E). Interestingly, METTL3 knockdown significantly reduced the relative expression of SUCLG2‐AS1 (Figure 3A,B). The expression of SUCLG2‐AS1 increased when METTL3 was overexpressed (Figure S3C). Further research suggested a positive correlation between METTL3 and SUCLG2‐AS1 in the TCGA‐HNSC database (Supplementary Figure S3D). Using SRAMP software (http://www.cuilab.cn/sramp), 14 reliable m6A modification sites on the SUCLG2‐AS1 transcript were predicted (Figure 3C). After treatment with the m6A synthesis inhibitor (cycloleucine), the expression of SUCLG2‐AS1 RNA decreased and was correlated with the concentration of cycloleucine (Figure 3D). We speculated that METTL3 might be involved in regulating the m6A modification of SUCLG2‐AS1 in NPC. Subsequently, MeRIP‐PCR analysis indicated that silencing of METTL3 attenuated the m6A modification of SUCLG2‐AS1 in NPC cells (Figure 3F,G). We then designed the m6A mutant (A–G) SUCLG2‐AS1 plasmid (Figure 3H). MeRIP‐qPCR showed that the m6A level in the SUCLG2‐AS1 mutant transcript was significantly lower than that in the wild‐type transcript of SUCLG2‐AS1 (Figure 3I,J). We further analyzed the half‐life of SUCLG2‐AS1 in METTL3‐silenced NPC cells by a single‐phase exponential decay model following actinomycin D treatment. METTL3 knockdown significantly decreased the half‐life of SUCLG2‐AS1 (Figure 3K,L). Silencing METTLE did not affect the expression of SUCLG2‐AS1 containing m6A mutant(A‐G) (Supplementary Figure S3F). These results suggested that METTL3 regulates the stability of SUCLG2‐AS1 throughm6A modifications. Considering that the effect of METTL3 on SUCLG2‐AS1 expression may depend on the mediation of m6A readers, common m6A reader proteins, including HUR, HNRNPA2B1, IGF2BP1, IGF2BP2, and IGF2BP3, were silenced in NPC cells, and knockdown efficiency was successfully validated (Figure S3B,R). IGF2BP3 knockdown significantly decreased the expression level of SUCLG2‐AS1 (Figure 3 M,N). Further research suggested a positive correlation between IGF2BP3 and SUCLG2‐AS1 in the TCGA‐HNSC database (Figure S3E). Simultaneously, biotinylated SUCLG2‐AS1, but not biotinylated antisense SUCLG2‐AS1, was found to be enriched for IGF2BP3 protein using RNA pull‐down analysis in 5−8F and CNE1 cells (Figure 3O). Subsequently, we observed that IGF2BP3 antibody‐precipitated SUCLG2‐AS1 was significantly reduced in NPC cells following METTL3 silencing (Figure 3P,Q). RNA stability assays showed that IGF2BP3 knockdown remarkedly decreased the half‐life of SUCLG2‐AS1 (Figure 3S,T). Not surprisingly, silencing IGF2BP3 did not affect the expression of SUCLG2‐AS1 containing m6A mutant (A–G) (Figure S3G). Our data suggest that the m6A reader IGF2BP3 recognizes the METTL3‐mediated m6A modification of SUCLG2‐AS1 and maintains its stability by preventing its degradation.

### 4.4 SUCLG2‐AS1 regulates cell metastasis in NPC by affecting SOX2

**FIGURE 4:**
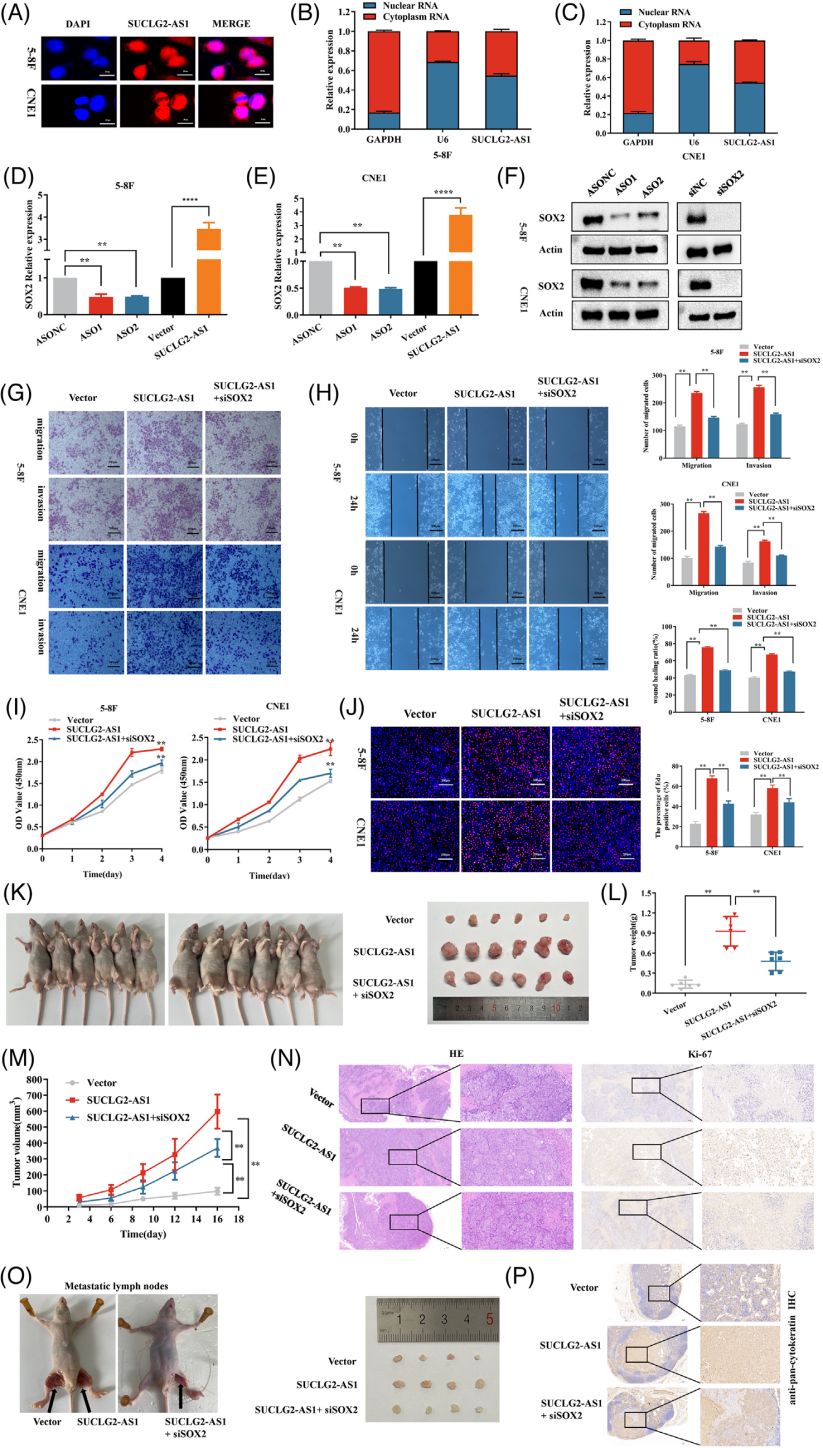
SUCLG2‐AS1 regulates cell metastasis of nasopharyngeal carcinoma (NPC) via affecting SOX2. (**A**) Fluorescent in situ hybridization (FISH) assays were applied to determine the subcellular localization of SUCLG2‐AS1 in NPC. (**B** and **C**) Nuclear and cytoplasmic separation assays were applied to determine the subcellular localization of SUCLG2‐AS1 in NPC.U6: nucleus control; GAPDH: cytoplasm control. (**D** and **E**) Knockdown and overexpression of SUCLG2‐AS1 to detect the expression of SOX2 via qRT‐PCR. (**F**) Knockdown of SUCLG2‐AS1 to detect the expression of SOX2 by western blot. (**G** and **H**) Wound healing and transwell assays showed that SUCLG2‐AS1 increased the cell invasion and migration of NPC through the promotion of SOX2 expression. (**I** and **J**) CCK8 and EDU assays identified that SUCLG2‐AS1 increased cell proliferation in NPC through the promotion of SOX2 expression. The results presented are from three independent experiments. (**K**) Representative images of xenograft tumours in three groups of nude mice. (**L** and **M**) Quantitative determination of weight and volume of xenograft tumours in three groups. n = 6 for each group. (**N**) H&E staining showed that the tumour was cancerous. IHC staining of Ki67 from xenograft tumor in situ. (**O**) Representative images of the metastatic popliteal lymph nodes in three groups. The number of lymph nodes metastasized in three groups. (**P**) IHC with anti‐pan‐cytokeratin antibody in the metastatic tumours of lymph nodes. Scale bar = 200 μm.*p < 0.05 and **p < 0.01.

To explore the potential mechanism of action of SUCLG2‐AS1 in NPC, we analyzed the subcellular localization of SUCLG2‐AS1 in CNE1 and 5−8F cells. Nuclear and cytoplasmic fractionation and fluorescent in situ hybridization showed that SUCLG2‐AS1 expression was slightly higher in the nucleus (Figure 4A–C), suggesting that SUCLG2‐AS1 may be involved in transcriptional regulation. A literature review found that SUCLG2‐AS1 is localized in the SEs region of SOX2, and 90% of SOX2 expression is driven by SEs.^12^ We thus determined the effect of SUCLG2‐AS1 on the expression of SOX2. Western blotting analysis showed that SUCLG2‐AS1 knockdown significantly suppressed SOX2 protein levels in 5−8F and CNE1 cells (Figure 4F), which was in agreement with the qRT‐PCR results (Figure 4D,E). SUCLG2‐AS1 overexpression increased SOX2 mRNA and protein expression. Compared with its expression in 10 normal tissues, SOX2 was significantly upregulated in N_2‐3_ stage NPC tumour tissues (Figure S4A). Further research suggested a positive correlation between SUCLG2‐AS1 and SOX2 in the TCGA‐HNSC database and 60 NPC patient samples (Figure S4B,C). 5−8F and CNE1 cells were transfected with siSOX2, and knockdown efficiency was successfully validated (Figure 4F and Figure S5A). Interestingly, siSOX2 restrained the proliferation, migration and invasion of NPC cells (Figure S5B–F). The efficiency of SOX2 overexpression was also evaluated (Figure S5G). Conversely, overexpression of SOX2 promoted migration and invasion of NPC cells (Figure S5H,I). As expected, the knockdown of SOX2 partially reversed the invasion, migration (Figure 4G,H) and proliferation (Figure 4I,J) effects of SUCLG2‐AS1 overexpression in NPC cells. In addition, we performed rescue experiments in vivo. As shown in Figure 4K–N, the tumours in the SUCLG2‐AS1 group were larger than those in the vector group. Furthermore, the knockdown of SOX2 partially reversed the proliferation of the tumours. Moreover, in the popliteal lymph node metastasis model, metastatic loci were increased in the SUCLG2‐AS1 overexpression group compared with those in the control group. And knockdown of SOX2 partially reversed the prometastatic ability mediated by SUCLG2‐AS1 (Figure 4O,P). These results suggest that SUCLG2‐AS1 regulates NPC cell proliferation and metastasis by affecting SOX2.

### 4.5 SUCLG2‐AS1 activates SOX2 transcription by binding to CTCF

**FIGURE 5:**
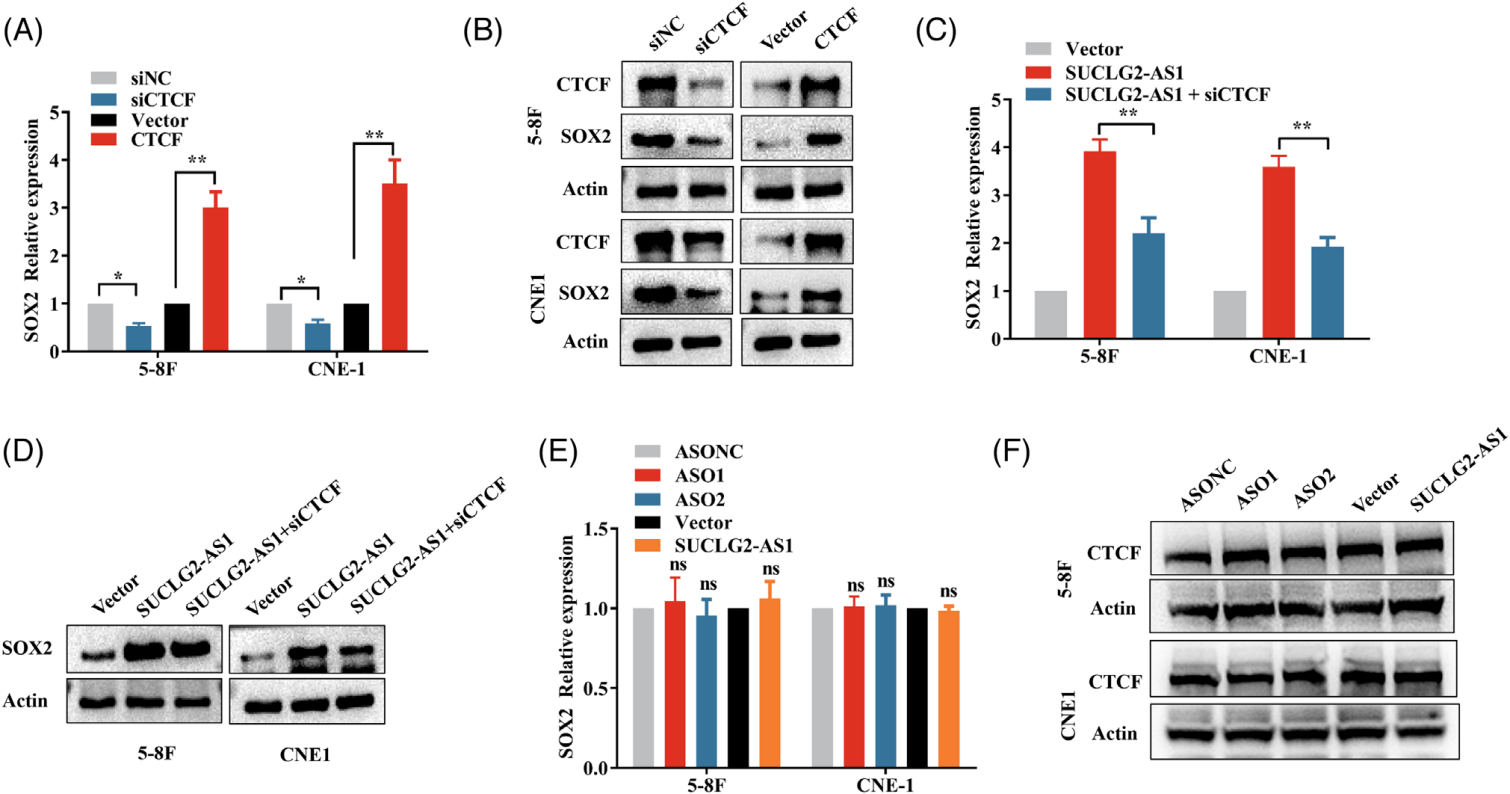
SUCLG2‐AS1 activates SOX2 transcription by binding to CTCF. (**A** and **B**) Relative SOX2 expression levels were determined by qRT‐PCR and western blot in CTCF knockdown and overexpression cells. (**C**) qRT‐PCR showed that SUCLG2‐AS1 reverses SOX2 expression of nasopharyngeal carcinoma (NPC) cells influenced by CTCF. (**D**) Western blot assays showed that SUCLG2‐AS1 reverses the SOX2 expression of NPC cells influenced by CTCF. (**E** and **F**) Relative CTCF expression levels were determined by qRT‐PCR and western blot in SUCLG2‐AS1 knockdown and overexpression cells. Relative mRNA expression levels were normalized to those of GAPDH. The results presented are from three independent experiments. siNC and siCTCF represent negative control and CTCF knockdown, respectively. *P < 0.05 and **p < 0.01. ns: not significant.

In this study, we interrogated the mechanism underlying the positive regulation of SOX2 by SUCLG2‐AS1 in NPC. SEs and their associated genes are located in the CTCF (CCCTC binding factor) loop in the genome. CTCFs are abundant in loop anchors and essential for loop formation and maintenance.^8^, ^16^, ^17^, ^18^ Bioinformatics analysis and annotation data from ENCODE showed that SUCLG2‐AS1 along with the super‐enhancer and promoter regions of SOX2 were specifically enriched in CTCF binding sites, implying that CTCF might play a key role in mediating long‐range loop interactions between promoter and super‐enhancer. Cells were transfected with siCTCF and pcDNA‐CTCF plasmid, and knockdown/ overexpression efficiency was successfully constructed (Figure 5B and Figure S6A,B). We examined whether CTCF affected SOX2 expression. CTCF knockdown dramatically suppressed the expression of SOX2 in NPC cells, whereas CTCF overexpression significantly enhanced the transcription levels of SOX2 (Figure 5A,B). Furthermore, SUCLG2‐AS1‐induced SOX2 expression was rescued by CTCF depletion (Figure 5C,D). We further explored whether SUCLG2‐AS1 affected CTCF expression. RT‐qPCR and western blotting revealed that the overexpression and silencing of SUCLG2‐AS1 had no influence on CTCF mRNA and protein expression (Figure 5E,F). Not surprisingly, the downregulation of CTCF attenuated NPC cell invasion, migration and proliferation (Figure S6C–F).

**FIGURE 6:**
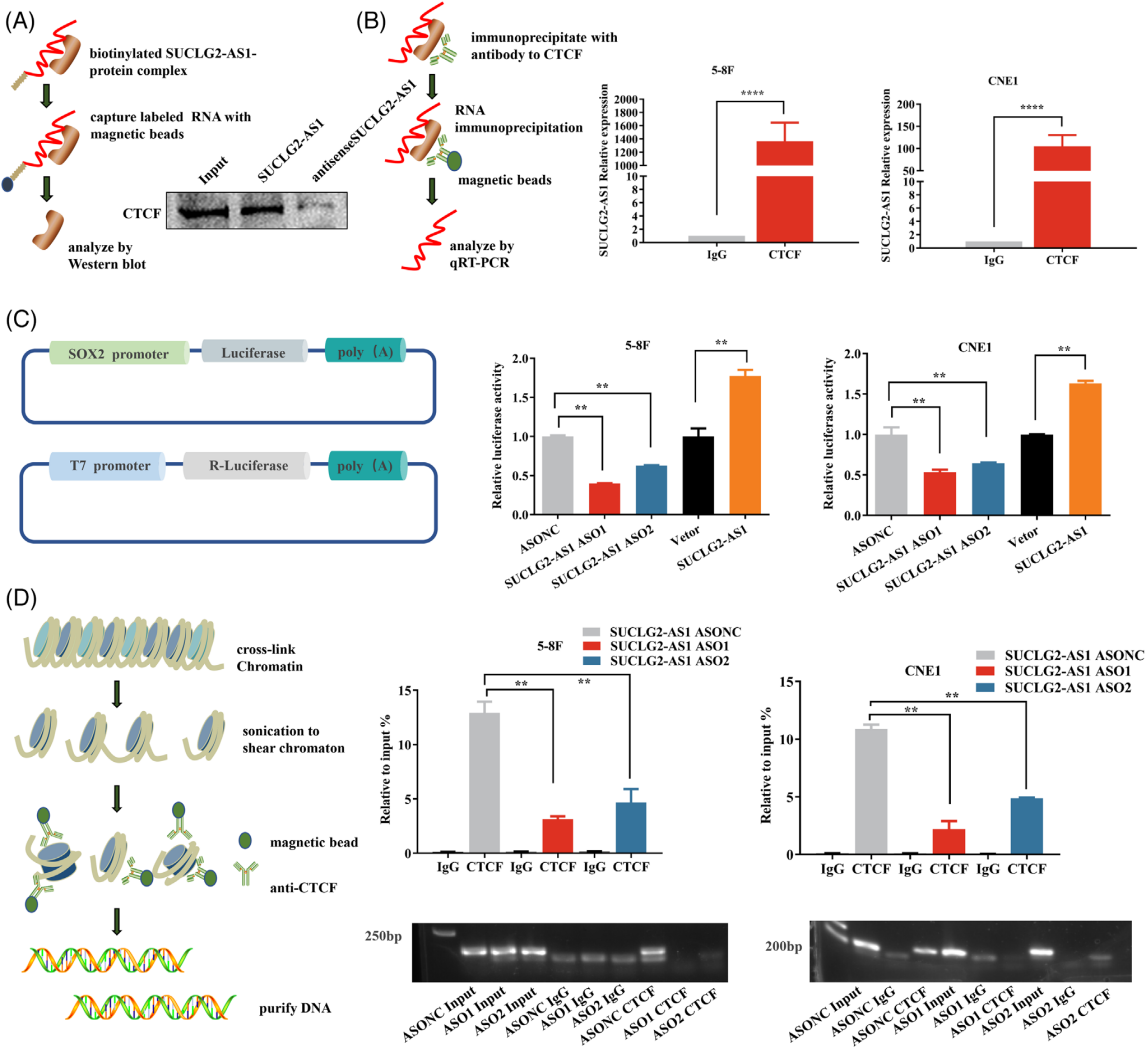
SUCLG2‐AS1 regulates the expression of SOX2 via mediating the CTCF‐occupied promoter region of SOX2. (**A**)RNA pull‐down assays of biotinylated SUCLG2‐AS1 special binding proteins were carried out. Western blot was conducted to determine the specific interaction of sense SUCLG2‐AS1 with CTCF. SUCLG2‐AS1‐antisense transcripts served as the control. (**B**) RIP assays were performed using anti‐CTCF antibody and control IgG. RNA immunoprecipitations were quantified by qRT‐PCR. (**C**) The luciferase reporter assays identified SOX2 promoter activity influenced by SUCLG2‐AS1. (**D**) ChIP‐qPCR was conducted on SOX2 promoter regions using CTCF antibodies in knockdown SUCLG2‐AS1 cells. Enrichment was determined relative to input controls. The results presented are from three independent experiments. *p < 0.05 and **p < 0.01.

To gain insight into the CTCF sites of SUCLG2‐AS1, a proteomic approach was used to determine the protein‐binding partners of in vitro biotinylated SUCLG2‐AS1 transcript in CNE1 cells. We confirmed that biotinylated SUCLG2‐AS1 RNA rather than antisense SUCLG2‐AS1 bound to CTCF (Figure 6A). Moreover, RIP assays confirmed the abundance of SUCLG2‐AS1 in CTCF immunoprecipitates from 5−8F and CNE1 cell lysates (Figure 6B). This suggested that SUCLG2‐AS1 interacts with CTCF in NPC cells. We further explored whether SUCLG2‐AS1 could trans‐activate the downstream target SOX2 through direct interaction with its promoters via CTCF. We performed a luciferase reporter assay to determine whether overexpression or knockdown of SUCLG2‐AS1 has an effect on the activity of the SOX2 promoter‐reporter. SUCLG2‐AS1 knockdown significantly attenuated the activity of the SOX2 promoter, whereas overexpression of SUCLG2‐AS1 increased the activity (Figure 6C). We used an anti‐CTCF antibody to immunoprecipitate the DNA‐protein complex from cells transfected with SUCLG2‐AS1 ASO and then used primers specific to this region of the SOX2 promoter for PCR amplification. Subsequent results from the chromatin immunoprecipitation assay showed that SUCLG2‐AS1 knockdown reduced CTCF occupancy in the promoter and, in turn, hindered the expression of SOX2 (Figure 6D). Overall, SUCLG2‐AS1‐mediated SOX2 activation was dependent on CTCF.

### 4.6 SUCLG2‐AS1 regulates the expression of SOX2 via long‐range chromatin loop formation, which via mediating CTCF occupied the SE and promoter region of SOX2

**FIGURE 7:**
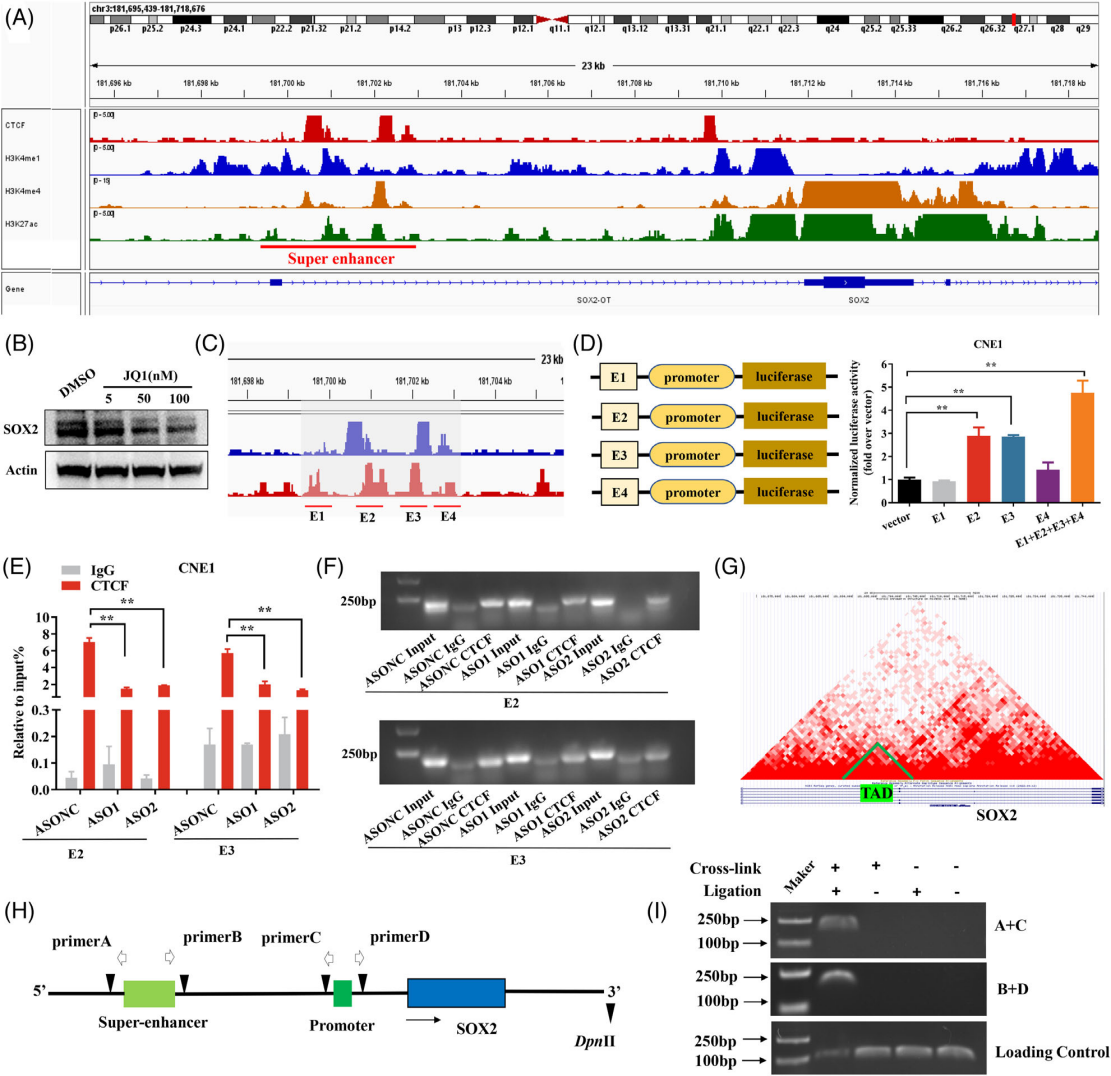
SUCLG2‐AS1 regulates expression of SOX2 via long‐range chromatin loop formation, which via mediating CTCF occupied super‐enhancer and promoter region of SOX2. (**A**) Integrated Genome Viewer (IGV) screenshot shows the ChIP‐seq signals of H3K27Ac, H3K4me1, H3K4me4 and CTCF at the SOX2 locus. The super‐enhancer region is highlighted in red. (**B**) Western blot detected SOX2 expression after treatment with different concentrations of JQ1 inhibitors. DMSO was the control treatment. (**C**) The super‐enhancer was divided into four segments (E1, E2, E3 and E4). (**D**) The luciferase reporter assays indicated the activities of E1, E2, E3 and E4. (**E** and **F**) ChIP‐qPCR was conducted on E2 and E3 regions using CTCF antibodies in SUCLG2‐AS1‐silenced cells. Enrichment was determined relative to input controls. (**G**)The location of super‐enhancer and promoter of SOX2 with Hi‐C interaction data from ENCODE. (**H**) 3C analysis of the interaction between SOX2 super‐enhancer and promoter region. (**I**) The upper and middle bands produced by the ligation products indicated that the SOX2 super‐enhancer was linked with the promoter; the PCR product of the bottom panel that was not cleaved by any restriction enzyme was used as the loading control. *p < 0.05 and **p < 0.01.

We further explored whether SUCLG2‐AS1 could trans‐activate the downstream target SOX2 through direct interaction with its enhancers by mediating CTCF. Integrated Genome Viewer (IGV) displayed images of the SOX2 locus in the genome‐wide ChIP‐sequencing dataset. The ChIP‐seq signals for H3K27Ac, H3K4me1, H3K4me4 and CTCF are shown in Figure 7A. SE is a large cluster of enhancers with transcriptional activity that are enriched with high concentrations of master transcription factors, cofactors and histone modification marks.^19^ The BRD4 inhibitor, JQ1(a small molecule epigenetic inhibitor, from MedChemExpress), competes with acetylated residues to bind to the bromine domain of BRD4, releasing BRD4 from chromatin and breaking down the interaction between SE and the promoter, decreasing RNA‐Pol II flux and intercepting the transcription of key oncogenes.^20^ We used JQ1 inhibitors to treat CNE1 cells and examined SOX2 expression by western blotting. We found that as the dose of JQ1 increased, SOX2 expression decreased (Figure 7B). To further evaluate enhancer activity, we evaluated the activity of SOX2 enhancers in NPC. The super‐enhancer of SOX2 was further divided into four components (E1–4) (Figure 7C), which were constructed separately or together in a pGL3 promoter vector. Luciferase assays were performed using the promoter regions of SOX2 fused to firefly luciferase. Importantly, compared to the negative control, the enhancer activity of E1+E2+E3+E4 in CNE1 cells was significantly increased, especially that of E2 and E3 (Figure 7D). ChIP‐qPCR assays showed that CTCF binds to the SOX2 enhancer domain. We determined whether SUCLG2‐AS1 influences the binding between CTCF and SOX2 enhancers. Notable, SUCLG2‐AS1 silencing increased the occupancy of CTCF in the SOX2 enhancer region (Figure 7E,F). Moreover, Hi‐C data predicted that there were significant physical interactions between the SOX2 promoter and super‐enhancer (Figure 7G). Considering the physical distance between these regulatory elements, we performed chromosome conformation capture analysis in NPC cell lines to detect long‐term chromatin interactions in vivo. A. B, C and D primers were used to detect the DNA sequences of ligation and non‐crosslinking after digestion or indigestion by restriction enzymes (Figure 7H). The PCR products revealed a long‐distance physical interaction between the super‐enhancer within 50 kb upstream of SOX2 and the promoter region of the SOX2 gene (Figure 7I). In summary, SUCLG2‐AS1 increased the occupancy rate of CTCF in the SOX2 promoter and enhancers, enhanced transcription and induced the accumulation of SOX2.

### 4.7 SUCLG2‐AS1 regulates NPC radiosensitivity via SUCLG2‐AS1/CTCF/SOX2 axis

**FIGURE 8:**
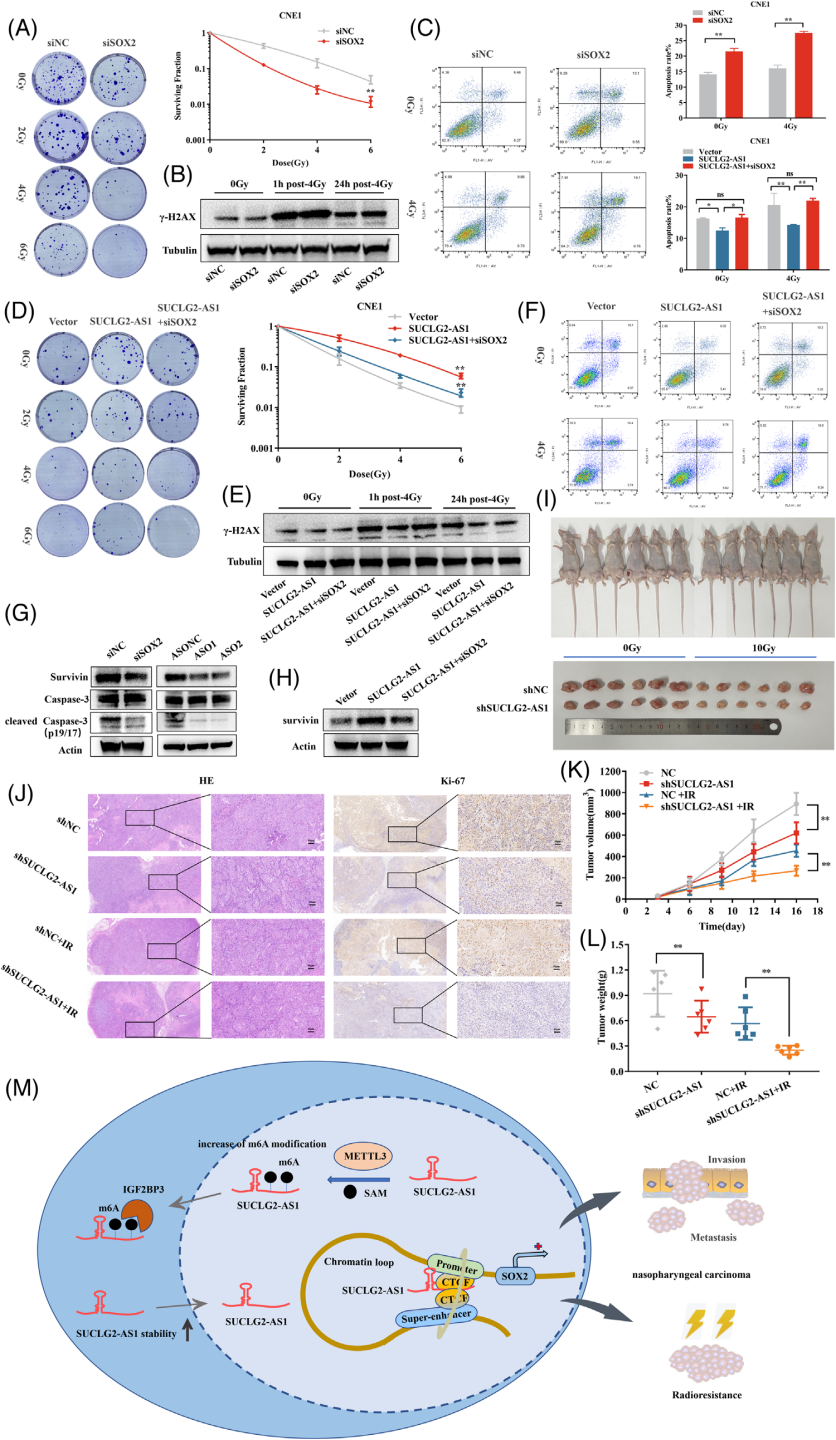
SUCLG2‐AS1 regulates nasopharyngeal carcinoma (NPC) radiosensitivity via SUCLG2‐AS1/CTCF/SOX2 axis. (**A**) Radiation survival assay showed that transfection of siSOX2 decreased NPC cells' radioresistance compared with the control group after 0, 2, 4 and 6 Gy irradiation. *P* < 0.05, two‐way analysis of variance (ANOVA) for data analysis. (**B**) Western blot analysis was performed on the expression of γ‐H2AX in CNE1 cells transduced with siSOX2 at 1 or 24 h after 4 Gy irradiation. (**C**) CNE1 cells were transfected with siNC or siSOX2 and then subjected to PI staining before flow cytometry. Quantitative and statistical analysis of the apoptosis rate is shown. (**D**) Radiation survival assay shows that overexpression SUCLG2‐AS1 reversed radioresistance which decreased by siSOX2 after 0, 2,4 and 6 Gy irradiation. (**E**) Western blot analysis was performed on the expression of γ‐H2AX in CNE1 cells transduced with siSOX2 and overexpression SUCLG2‐AS1 at 1 or 24 h after 4 Gy irradiation. (**F**) Flow cytometry analysis showed that overexpression of SUCLG2‐AS1 reversed apoptosis rate which increased by siSOX2. (**G**) Western blot analysis showed that apoptosis‐related protein was present in CNE1 cells after SOX2/SUCLG2‐AS1 silencing. (**H**) Western blot analysis showed that survivin protein was also observed in CNE1 cells after co‐transfecting siSOX2 and SUCLG2‐AS1 overexpression. (**I**) Representative images of xenograft tumors in four groups of nude mice with or without irradiation. (**J**) H&E staining showed that the tumour was cancerous. IHC staining of Ki67 from xenograft tumour in situ. (**K**) Quantitative determination of the volume of xenograft tumours in four groups. n = 6 for each group. (**L**) Quantitative determination of the weight of xenograft tumours in four groups at the end of the research. The results presented are from three independent experiments. *p < 0.05 and **p < 0.01. (**M**) Graphical scheme of METTL3‐stabilized SE‐lncRNA SUCLG2‐AS1 mediates long‐range chromatin interactions between enhancers and promoter of SOX2.

SOX2 is a marker of cancer stem cells that is highly expression in response to radiotherapy.^21^ We speculate that SUCLG2‐AS1 mediates radiotherapy resistance in NPC through SOX2. We exposed SOX2‐depleted CNE1 cells to different radiation doses (0, 2, 4 and 6 Gy) and measured clonogenic cell survival. The data showed that compared with the untreated control, SOX2 knockdown significantly decreased the number of colonies formed in a dose‐dependent manner when cells were irradiated (Figure 8A); SUCLG2‐AS1 knockdown showed the same effect (Figure S7A). We found that irradiation remarkably induced DNA damage maker γ‐H2AX expression in SOX2‐depleted cells compared to control counterparts (Figure 8B). Furthermore, SUCLG2‐AS1 knockdown increased levels of γ‐H2AX at 1 h after irradiation (Figure S7C). Importantly, we found that SOX2 knockdown significantly increased apoptosis after exposure of cells to 4 Gy of radiation (Figure 8C), which was consistent with the results of SUCLG2‐AS1 knockdown (Figure S7B). Co‐transfection SUCLG2‐AS1 overexpression and siSOX2 into CNE1 cell showed that decreased SOX2 expression abrogated the remarkable improvement in radiosensitivity by upregulating SUCLG2‐AS1 expression (Figure 8D). After exogenous overexpression of SUCLG2‐AS1, γ‐H2AX significantly decreased at 1 h after irradiation and was rescued by SOX2 knockdown (Figure 8E). SOX2 depletion significantly reversed the irradiation‐induced apoptotic response to SUCLG2‐AS1 overexpression in CNE1 cells compared with that in control cells (Figure 8F). We also detected variations in the master factors of apoptosis signalling pathways. Furthermore, SOX2 knockdown increased the expression of cleaved caspase‐3 and survivin. Consistent with this finding, SUCLG2‐AS1 knockdown resulted in increased expression of cleaved caspase‐3 and survivin (Figure 8G). Co‐transfection with pcDNA‐SUCLG2‐AS1 and siSOX2, partially restored survivin expression (Figure 8H). The implies that survivin may direct the contribution of SOX2 to DNA damage. Furthermore, we constructed xenograft model stable cell lines of shSUCLG2‐AS1 (Figure 8I). The results demonstrated that both the volume and weight of tumours in the shSUCLG2‐AS1 group were markedly smaller than those in the shNC group. Furthermore, the volume and weight of the tumours were drastically smaller in the shSUCLG2‐AS1 irradiated group than in the non‐irradiated group (Figure 8K,L). SOX2 expression significantly decreased in the subcutaneous tumours of the SUCLG2‐AS1 knockdown group (Figure S8A). Hematoxylin and eosin‐staining revealed that the tumor was cancerous. IHC staining showed that SUCLG2‐AS1 knockdown tumors presented decreased expression of the proliferation marker Ki67 (Figure 8J). These results indicate that SUCLG2‐AS1 regulates the radiosensitivity and metastasis of NPC cells via SOX2. (Figure 8M)

## 5 DISCUSSION

With the popularity of intensity‐modulated radiotherapy (IMRT), the local control rate of NPC has reached approximately 90%; however, distant metastasis remains the principal cause of treatment failure. At the molecular level, NPC metastasis is a complex biological process, determined by the activity of the gene regulatory network, which is regulated by many core genes.^22^, ^23^ In the past decade, the discovery of a majority of lncRNAs has dramatically changed our understanding of tumorigenesis. Long non‐coding RNA participates in NPC metastasis at multiple levels.^24^ Remarkably, SE‐lncRNAs have gradually been discovered. To provide new ideas for a broad view of the regulatory mechanism of NPC invasion and metastasis, we explored novel targets of SE‐lncRNA in NPC. In addition, the systematic screening of lncRNAs associated with NPC metastasis is lacking. This prompted us to search for SE‐lncRNA associated with NPC metastasis NPC.

Richard A. Young proposed a SE based on enhancers.^25^ Enhancers are non‐coding DNA cis‐acting elements in the genome. SEs are clusters of enhancers that have a large content of genomic regulatory elements, occupied by aberrant high enrichment master transcription factors (TFs), active histone marks (H3K4me, H3K27ac), and transcription regulator factors (Mediator, BRD4, Pol II and p300).^19^, ^25^, ^26^ Because of the combination with the transcription mechanism, the active enhancer region is generally transcribed into non‐coding RNA, or the trans‐non‐coding RNA interacts with the enhancer region, both of which have a role in the local enhancement of biological activity in some cases. Several recent studies have suggested that SE‐lncRNAs regulate the activity of SEs. SE‐lncRNAs have been shown to exert enormous function on tumorigenesis. The transcription factor TP63 binds to the SEs region where LINC01503 is located to enhance its transcription and facilitate the invasion and metastasis of head and neck squamous cell carcinoma.^27^ These results demonstrate the importance of SE‐lncRNAs in the modulation of gene expression and tumour metastasis. Using sequencing data from the SE‐lncRNA database, we identified a previously unknown metastasis‐associated lncRNA, SUCLG2‐AS1 (Ensembl ID: ENSG00000227533). The sequence of SUCLG2‐AS1 was detected by RACE, and the full‐length SUCLG2‐AS1 was 1984 nt.

With the in‐depth study of m6A modification, the potential impact of m6A on lncRNAs had attracted considerable attention.^28^ Emerging evidence suggests that the m6A writer METTL3 enhances ncRNA stability by mediating m6A modification of ncRNAs.^29^ In addition, IGF2BP3, one of the most common m6A readers, recognizes m6A modifications in mRNA or ncRNAs and promotes their stability.^30^ In our study, SUCLG2‐AS1 was written into m6A by METTL3 in the nucleus, and SUCLG2‐AS1 was recognized and read by IGF2BP3 in the cytoplasm to induce the expression of SUCLG2‐AS1, and then SUCLG2‐AS1 entered the nucleus to play its role. Our study provides new evidence for the involvement of m6A modifications in lncRNA metabolism.

SUCLG2‐AS1 is located in the SEs region of SOX2. The sex‐determining region Y‐box 2 (SOX2) is one of the SRY‐related HMG‐box (SOX) transcription factor family and contains a unique high mobility group (HMG) domain that binds DNA in a sequence‐specific way.^31^ Studies have confirmed that deleting the distal SE of SOX2 diminishes its expression by more than 90%.^32^, ^33^ In ES cells, SOX2 transcription is regulated by distal enhancers via contact with the proximal region of the SOX2 promoter.^34^ Evidence suggests that SOX2 could upregulate β‐catenin signalling, thus regulating metastasis of nasopharyngeal carcinoma.^35^ In lung cancer and prostate cancer, survivin was a key downstream molecule of SOX2 in anti‐apoptosis.^36^, ^37^, ^38^ The relationship between SOX2 and survivin was also confirmed in our research.

Our results showed that SUCLG2‐AS1, together with the enhancer and promoter regions of SOX2, was enriched at the CTCF‐binding sites, suggesting that SOX2 is expressed under the regulation of transcription factors. CTCF (CCCTC‐binding factor) is a multifunctional transcription factor.^39^ CTCF binds to DNA sequences or proteins to regulate gene expression.^40^ Cancer‐specific CTCF binding promotes progressive and metastatic transcriptional dysregulation, not only promoting the formation of higher‐order chromatin structures but also promoter activation and repression.^41^ Downregulation of CTCF may disrupt the loop structure and reduce target gene expression. Studies have shown that SEs and their associated genes are often located in the CTCF loop of the genome.^33^ We further explored whether CTCF affects SOX2 expression by interacting with the promoter and super‐enhancer regions and serves as an anchor to recruit co‐activators for SOX2 transcription. In line with our hypothesis, CTCF occupied super‐enhancer and promoter regions that regulated SOX2 expression, which was mediated by SUCLG2‐AS1.

SE‐lncRNAs, act as scaffold molecules that direct various factors to their target sites to regulate gene expression. Specifically, SE‐lncRNAs regulate distant gene expression by interacting with promoters and enhancers or their binding proteins by affecting the chromatin state and RNA polymerase activity. For example, the lncRNA HoxBlinc activates NPM1c+ genes by enhancing enhancer/promoter chromatin accessibility in HSPCs.^42^ Pcdha‐as promotes demethylation of the CTCF‐binding site upstream of the Pcdha PCG and forms a stable loop with the distal enhancer region to positively regulate PCG expression.^43^ Intriguingly, SE‐lncRNAs can form a loop domain through the transcription factor CTCF and the promoter of the target gene, thereby remotely enhancing the transcription of the target gene.^8^, ^44^ This structure forms a loop between the gene promoter and distal regulatory elements to recruit a variety of transcription factors to promote gene expression. The promoter regions of SOX2 and SUCLG2‐AS1 were both enriched in CTCF binding sites, and our research showed that SUCLG2‐AS1 could transactivate the downstream target SOX2 through direct interaction with its promoters. SUCLG2‐AS1 increased the occupancy rate of CTCF in the SOX2 promoter and enhancers, enhanced transcription and induced the accumulation of SOX2. Specifically, this is the first time that we found that SE‐lncRNA SUCLG2‐AS1 is linked to the tumour metastasis driver gene CTCF, contributing to SOX2 through the loop regulation ability to form RNA/DNA/DNA triplexes in NPC.

Radioresistance of tumour cells remains a major problem owing to the poor efficacy of radiotherapy and chemotherapy. Radiosensitivity is affected by many factors, such as apoptosis, cell cycle, DNA damage and repair.^45^, ^46^ Previous studies have found that high expression of SOX2 in tumours could mediate radiotherapy resistance in NPC.^47^, ^48^ Our results showed that the SUCLG2‐AS1/CTCF/SOX2 axis regulates NPC radiosensitivity. Several studies have shown that lncRNAs regulate radiosensitivity.^49^, ^50^ This is the first report of the effect of SUCLG2‐AS1 on the radiosensitivity of NPC; SUCLG2‐AS1 is a novel lncRNA closely related to the radiosensitivity of NPC.

Taken together, our data suggest that SUCLG2‐AS1 serves as an oncogene in NPC and, is correlated with lymph node metastasis and poor prognosis. SUCLG2‐AS1 recruits CTCF, which could mediate the interaction of SE of SOX2 with the promoter region, remotely enhancing the transcriptional activation of SOX2 and, promoting the invasion, metastasis and radioresistance of NPC. Our data provide novel therapeutic indicators and latent targets for NPC treatment.

## CONFLICT OF INTEREST STATEMENT

The authors declare no conflict of interest.

## FUNDING INFORMATION

This study was supported by the National Natural Science Foundation of China (81802701, 82002869 and 81872192), China International Medical Foundation (NTC03932266) and the Key Program of Jiangsu Provincial Department of Science and Technology (BE2019756).

## Supporting information

Supporting information

Supporting information
