# Supplementary material for: METTL3‐stabilized super enhancers‐lncRNA SUCLG2‐AS1 mediates the formation of a long‐range chromatin loop between enhancers and promoters of SOX2 in metastasis and radiosensitivity of nasopharyngeal carcinoma
Source: Clin Transl Med. 2023 Sep 1;13(9):e1361. doi: 10.1002/ctm2.1361 (PMC10474317; doi:10.1002/ctm2.1361)
Supplement: Supplementary file 1 — Supporting information [file CTM2-13-e1361-s002.docx]

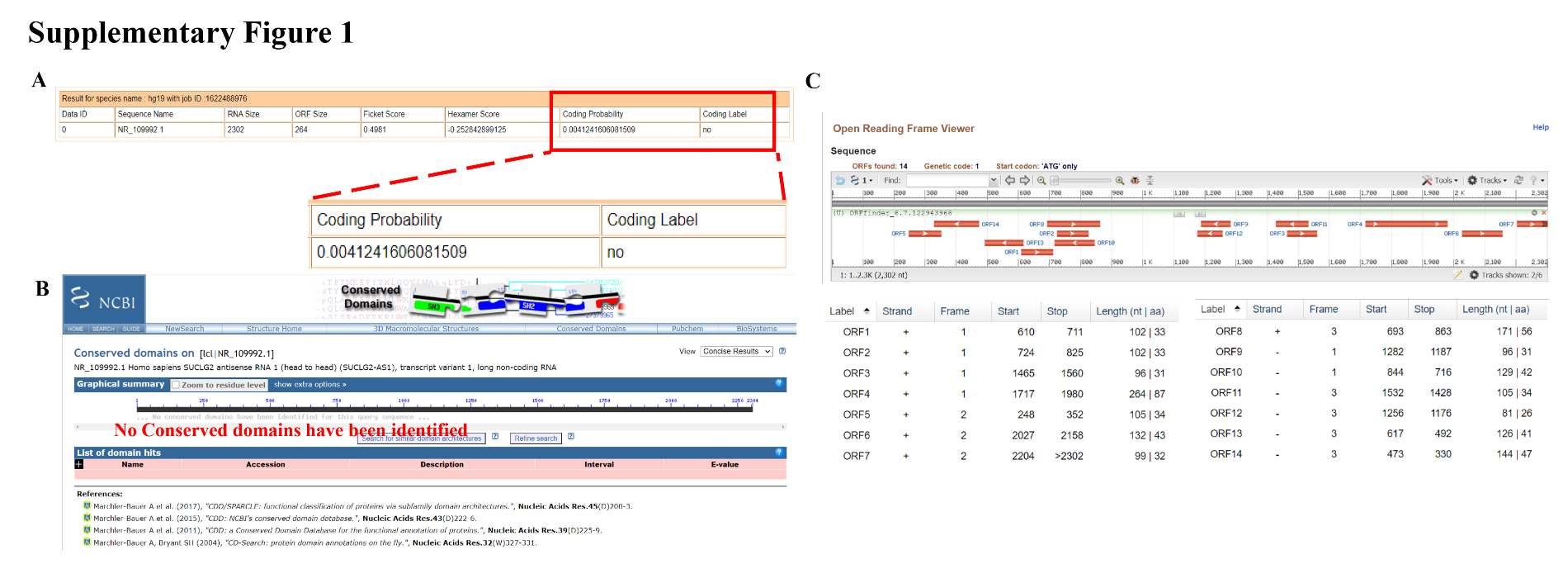


**Supplementary Figure 1**

(**A-C**) UCSC database screenshot shows that SUCLG2-AS1 has no coding potential.


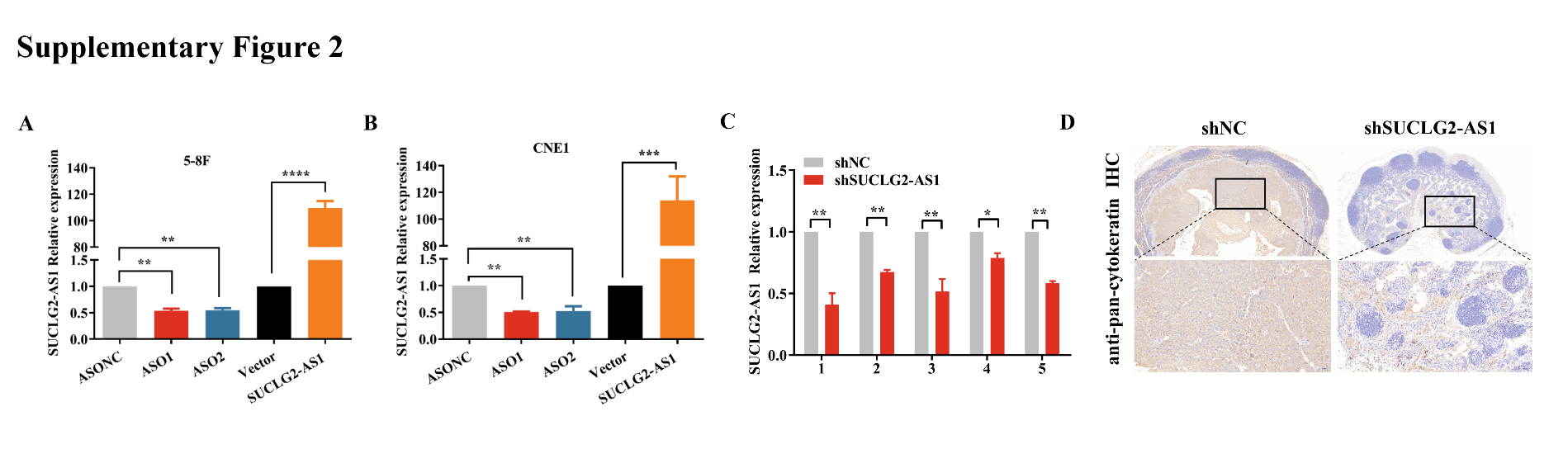
**Supplementary Figure 2**

(**A** and **B**) The efficiency of the interference and overexpression of SUCLG2-AS1 was detected via qRT-PCR. (**C**) SUCLG2-AS1 expression in the mice metastatic lymph nodes was detected via qRT-PCR. (**D**) IHC with anti-pan-cytokeratin antibody in the metastatic tumors of lymph nodes. **P* <0.05 and ***P* <0.01.

**
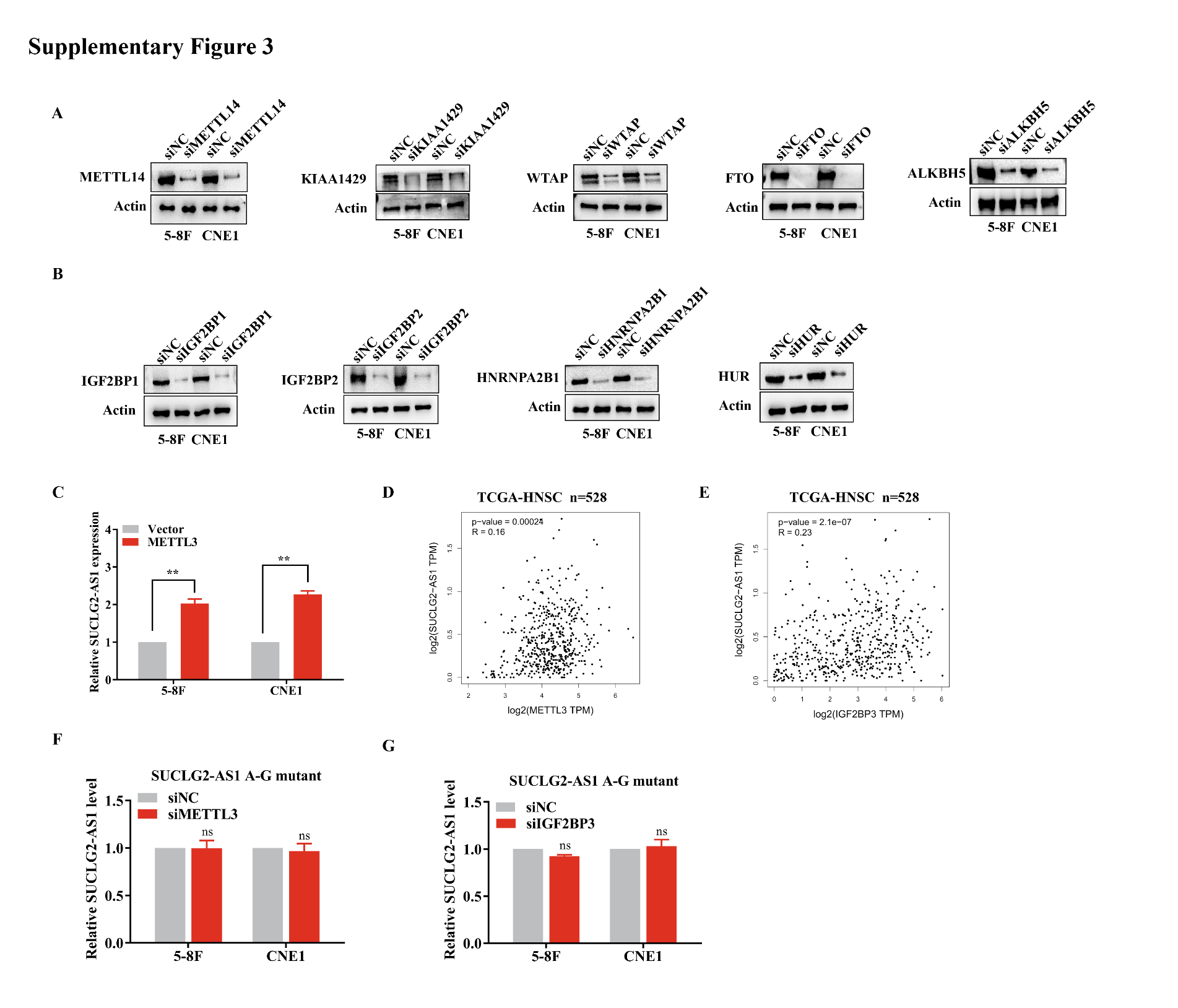
Supplementary Figure 3**

(**A**) Western blot was used to detect the efficiency of silencing METTL14, KIAA1429, WTAP, FTO and ALKBH5. (**B**) Western blot was used to detect the efficiency of silencing IGF2BP1, IGF2BP2, HUR and HNRNPA2B1. (**C**) qRT-PCR was used to detect SUCLG2-AS1 expression when METTL3 was overexpression. (**D**) Expression correlation of METTL3 and SUCLG2-AS1 in TCGA-HNSC database. (**E**) Expression correlation of IGF2BP3 and SUCLG2-AS1 in TCGA-HNSC database. (**F** and **G**) qRT-PCR was used to detect SUCLG2-AS1 expression in NPC cells transfected with SUCLG2-AS1 m6A mutant(A-G) plasmid and siMETTLE/siIGF2BP3. **P* <0.05 and ***P* <0.01.


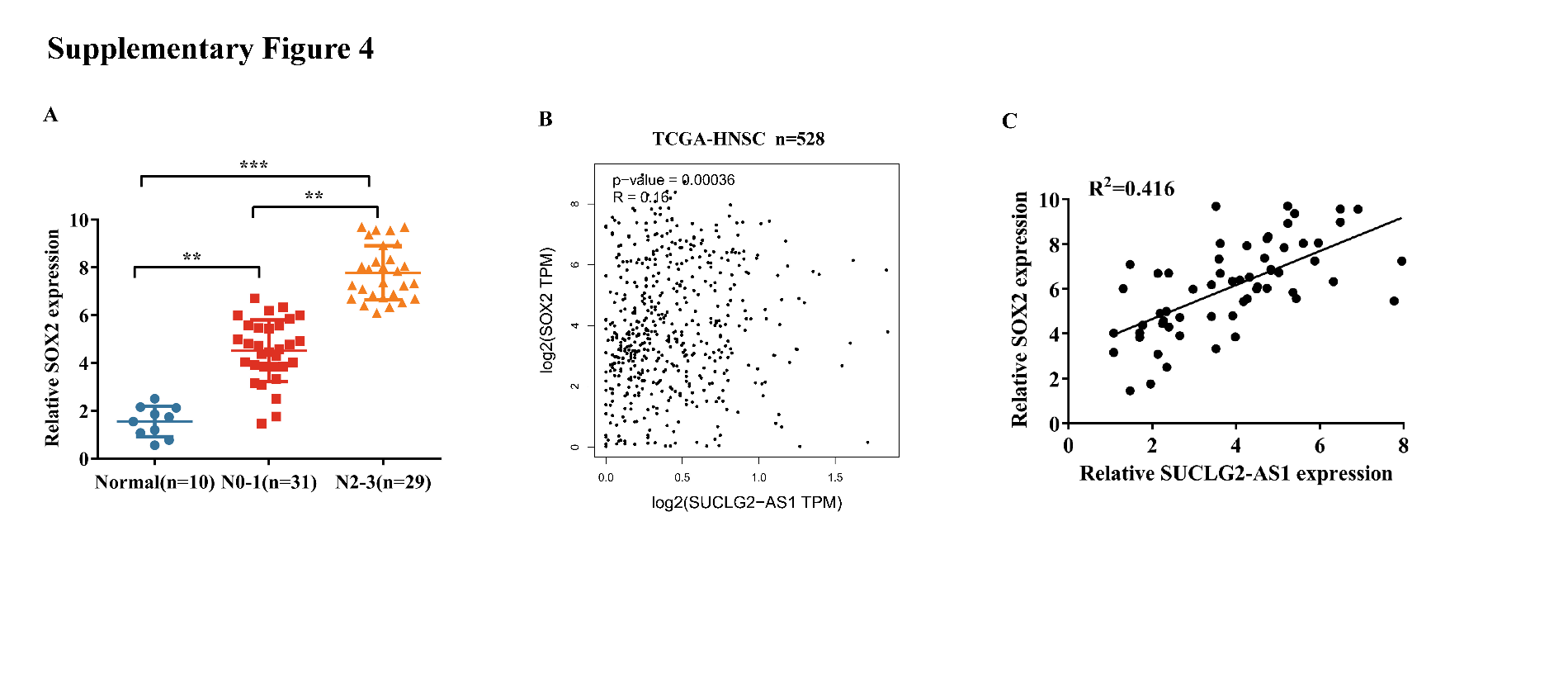
**Supplementary Figure 4**

(**A**) The expression of SOX2 was assayed in normal nasopharyngeal tissues and tumor tissues at N stages. (**B**) Expression correlation of SUCLG2-AS1 and SOX2 in TCGA-HNSC database. (**C**) Pearson correlation analysis between SUCLG2-AS1 and SOX2 in 60 NPC tissues. **P* <0.05 and ***P* <0.01.

**
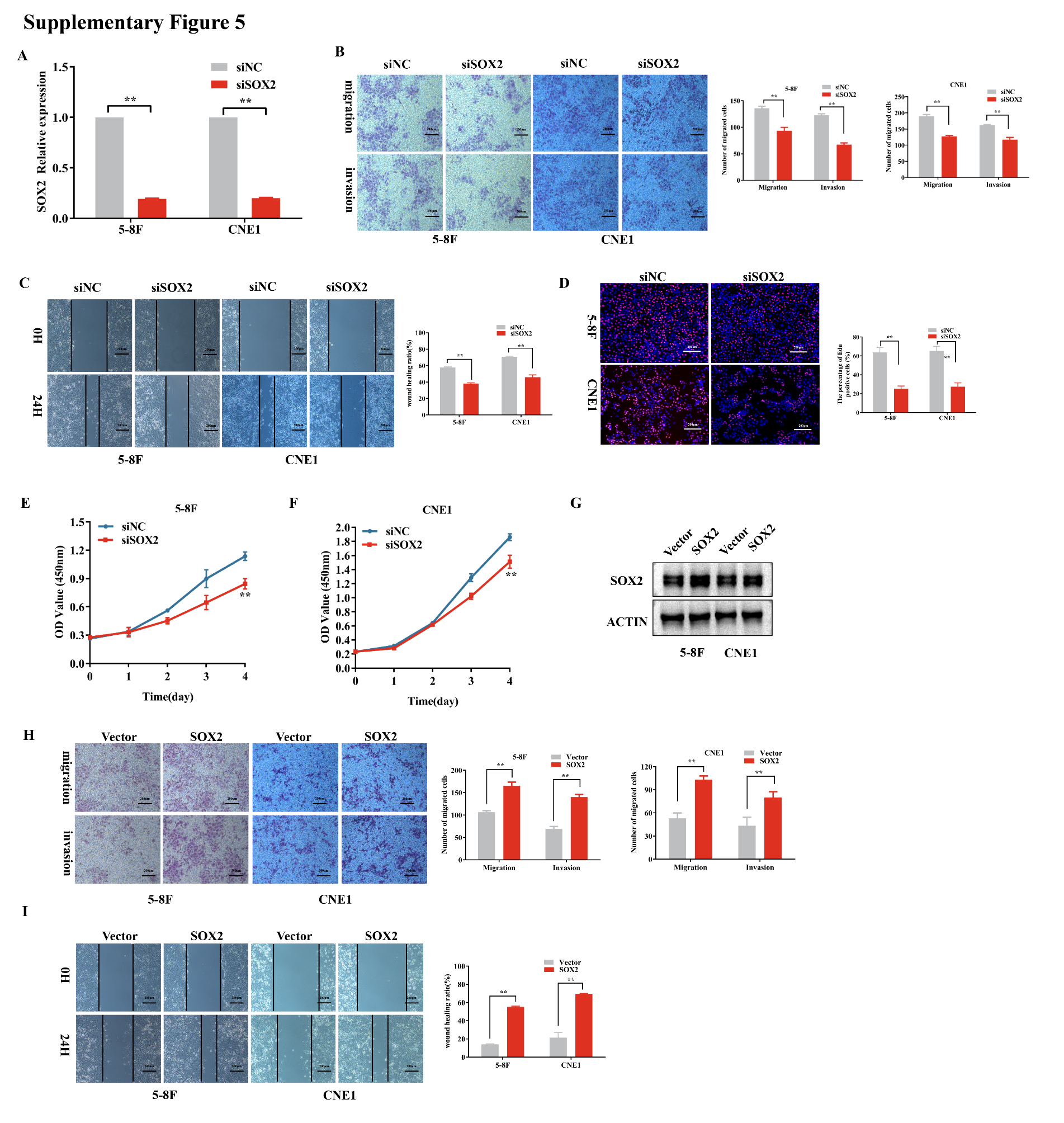
Supplementary Figure 5**

(**A**) Efficiency of knockdown of SOX2 was detected via qRT-PCR. (**B** and **C**) Transwell and wound healing assays were performed to determine the invasion and migration abilities of SOX2 knockdown cells. (**D, E** and **F**) The proliferation abilities of SOX2 knockdown cells were detected by EDU and CCK8 assays. (**G**) Efficiency of overexpression of SOX2 was detected via western blot. (**H** and **I**) Transwell and wound healing assays were performed to determine the invasion and migration abilities of SOX2 overexpression cells. **P* <0.05 and ***P* <0.01.


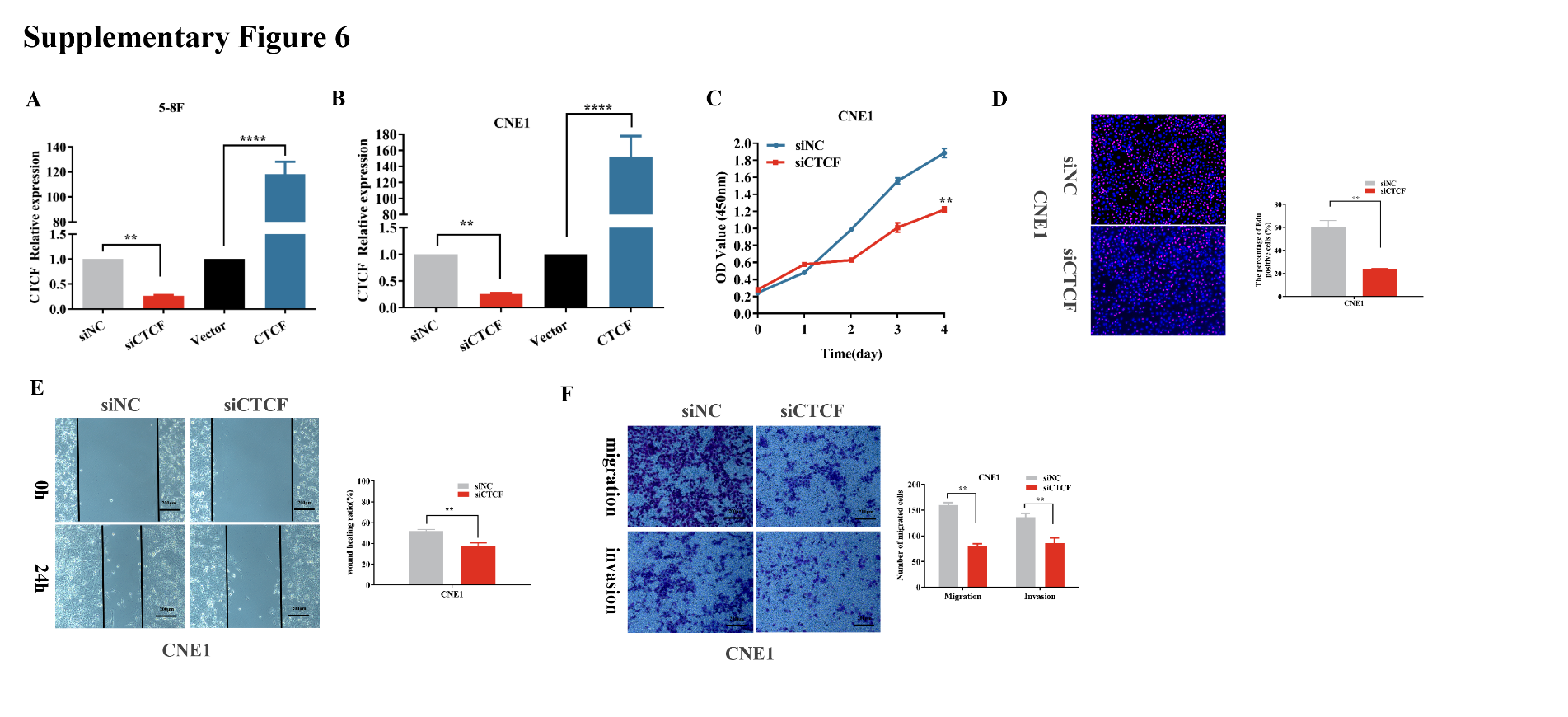
**Supplementary Figure 6**

(**A** and **B**) Efficiencies of the knockdown and overexpression of CTCF in NPC cells were detected via qRT-PCR. (**C** and **D**) CCK8 and EDU assays indicated that knockdown of CTCF decreased the proliferation of NPC cells. (**E** and **F**) Wound healing and transwell assays indicated that knockdown of CTCF decreased the invasion and migration of NPC cells. **P* <0.05 and ***P* <0.01.

**
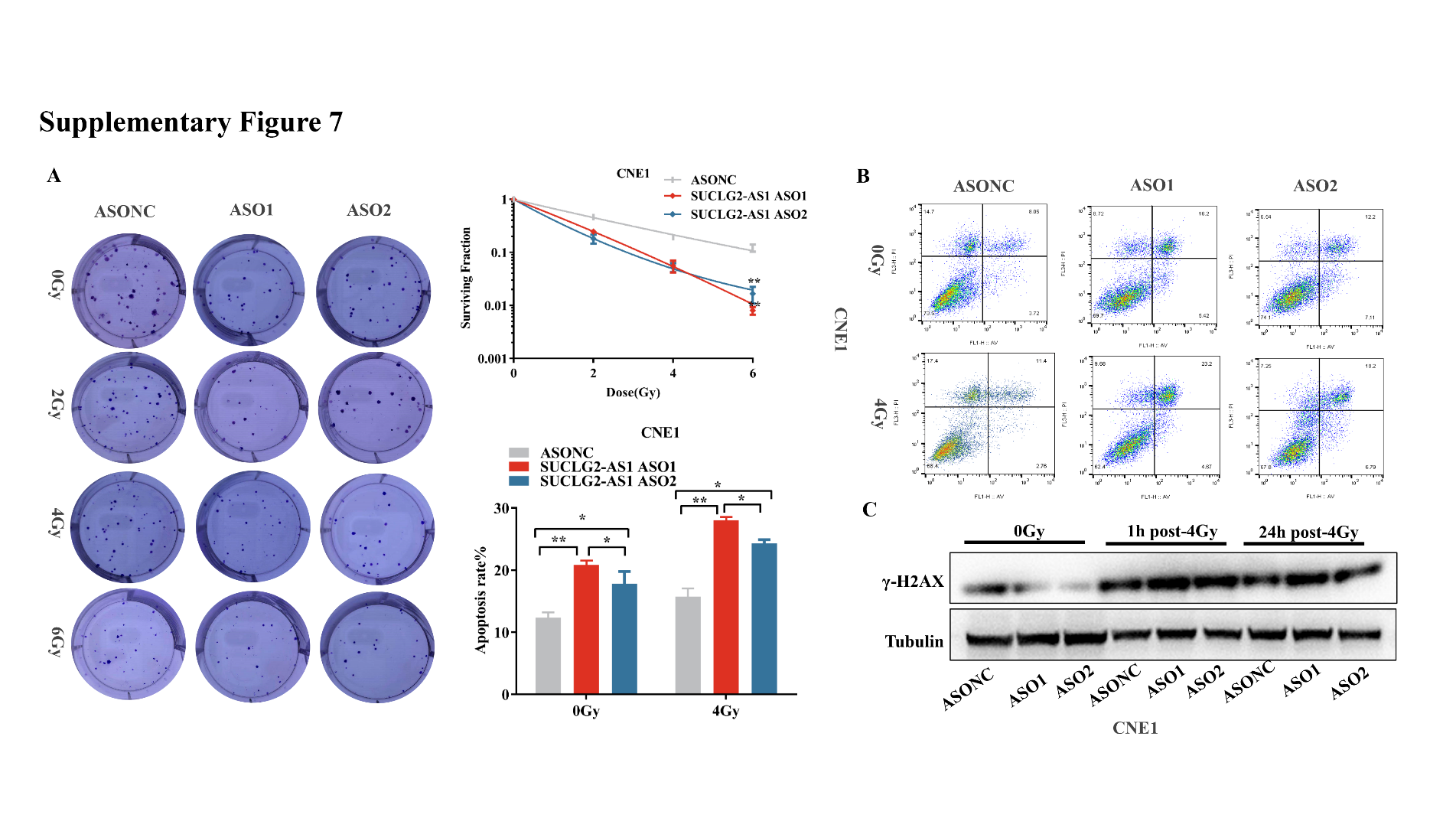
Supplementary Figure 7**

(**A**) Radiation survival assay showed that silencing SUCLG2-AS1 decreased NPC cells radioresistance compared with control group after 0, 2 ,4 ,6 Gy irradiation, two-way ANOVA for data analysis. (**B**) Flow cytometry analysis showed that silencing SUCLG2-AS1 decreased apoptosis rate with or without IR. (**C**) Western blot analysis was indicated the expression of γ-H2AX in CNE1 cells transduced with SUCLG2-AS1 ASO at 1h or 24h after 4 Gy IR. **P* <0.05 and ***P* <0.01.

**
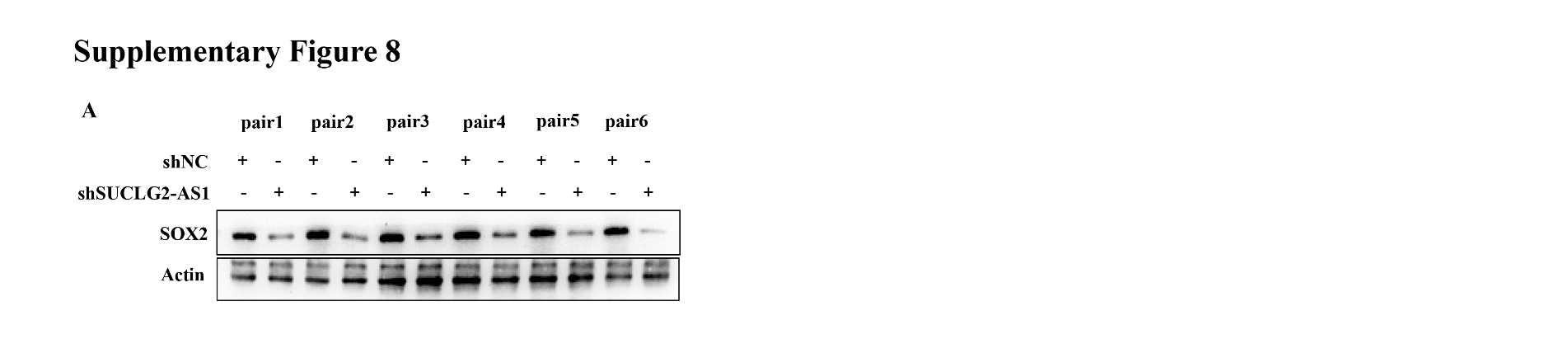
Supplementary Figure 8**

(**A**) Western blotting showed that SOX2 expression in the subcutaneous tumors of the SUCLG2-AS1 knockdown group and control group.
