# Supplementary material for: METTL3‐stabilized super enhancers‐lncRNA SUCLG2‐AS1 mediates the formation of a long‐range chromatin loop between enhancers and promoters of SOX2 in metastasis and radiosensitivity of nasopharyngeal carcinoma
Source: Clin Transl Med. 2023 Sep 1;13(9):e1361. doi: 10.1002/ctm2.1361 (PMC10474317; doi:10.1002/ctm2.1361)
Supplement: Supplementary file 2 — Supporting information [file CTM2-13-e1361-s001.docx]

**Summary of primers or siRNA sequences used**

| SUCLG2-AS1  RACE | Forward: GTCCCTGGATGGCCAAAC |
| --- | --- |
|  | Reverse: GGCACTTATCATGTGCCAGGTA |
| SUCLG2-AS1 | Forward: CCAGAAGGAAAAAGAGTGCTGT |
|  | Reverse: TGAGAAACTCCTGGTGATGGTT |
| CTCF | Forward: CAGTGGAGAATTGGTTCGGCA |
|  | Reverse: CTGGCGTAATCGCACATGGA |
| SOX2 | Forward: GCCGAGTGGAAACTTTTGTCG |
|  | Reverse: GGCAGCGTGTACTTATCCTTCT |
| GAPDH | Forward: CAATGACCCCTTCATTGACC |
|  | Reverse: TTGATTTTGGAGGGATCTCG |
| SOX2 promoter: | Forward: CCACATCCCTGAAACCAGCC |
|  | Reverse: AAGGTAAACACGGACCTGGA |
| E2 | Forward: TTTTGTGCTATCGCCTCCCC |
|  | Reverse: GAAAAGCTGAAGGATGGGGC |
| E3 | Forward: AAGCGAATCTACCAGCAGGG |
|  | Reverse: GATCTGGGGAAGAGAAGGCG |
| U6 | Forward: CTCGCTTCGGCAGCACA |
|  | Reverse: AACGCTTCACGAATTTGCGT |
| METTL3 | Forward: TTGTCTCCAACCTTCCGTAGT |
|  | Reverse: CCAGATCAGAGAGGTGGTGTAG |
| IGF2BP3 | Forward: TATATCGGAAACCTCAGCGAGA |
|  | Reverse: GGACCGAGTGCTCAACTTCT |

| siRNA | Targeted sequence |
| --- | --- |
| SUCLG2-AS1 ASO1 | AGGCGTTCTTAAGGCTGCAG |
| SUCLG2-AS1 ASO2 | GTTCTTAAGGCTGCAGAGCC |
| shSUCLG2-AS1 | CCTGTCACTCCTCCGTATT |

| siRNA | Sequence (5’-3’) |
| --- | --- |
| CTCF #1 | AGTTATGATTTCCCATCGA |
|  | TCGATGGGAAATCATAACT |
| CTCF #2 | CCATCAGTTAGCTATCAGA |
|  | TCTGATAGCTAACTGATGG |
| SOX2 #1 | CACCGGGCCCGCAGCAAGCTTG |
|  | CAAGCTTGCTGCGGGCCCGGTG |
| SOX2 #2 | AAACTTGTGGTGGCCGCAAAGC |
|  | GCTTTGCGGCCACCACAAGTTT |
| METTL3 #1 | GCUACCUGGACGUCAGUAUTT |
|  | AUACUGACGUCCAGGUAGCTT |
| METTL3 #2 | GGUUGGUGUCAAAGGAAAUTT |
|  | AUUUCCUUUGACACCAACCTT |
| METTL14 #1 | GGAUGAAGGAGAGACAGAUTT |
|  | AUCUGUCUCUCCUUCAUCCTT |
| METTL14 #2 | GCAGCACCUCGAUCAUUUATT |
|  | UAAAUGAUCGAGGUGCUGCTT |
| WTAP #1 | AAGCUUUGGAGGGCAAGUACA |
|  | UGUACUUGCCCUCCAAAGCUU |
| WTAP #2 | UUAAGAUCUGUGUACUUGCCC |
|  | GGGCAAGUACACAGAUCUUAA |
| FTO #1 | GCAGCUGAAAUACCCUAAATT |
|  | UUUAGGGUAUUUCAGCUGCTT |
| FTO #2 | AAAUAGCCGCUGCUUGUGAGA |
|  | UCUCACAAGCAGCGGCUAUUU |
| ALKBH5 #1 | ACAAGTACTTCTTCGGCGA |
|  | TCGCCGAAGAAGTACTTGT |
| ALKBH5 #2 | GCGCCGTCATCAACGACTA |
|  | TAGTCGTTGATGACGGCGC |
| IGF2BP1 #1 | CCAAAGUUCGUAUGGUUAUTT |
|  | AUAACCAUACGAACUUUGGTT |
| IGF2BP1 #2 | CCUGGCCCAUAAUAACUUUTT |
|  | AAAGUUAUUAUGGGCCAGGTT |
| IGF2BP2 #1 | GCGAAAGGAUGGUCAUCAUTT |
|  | AUGAUGACCAUCCUUUCGCTT |
| IGF2BP2 #2 | AGAGATTCAAGAGATCTCTTCG |
|  | CGAAGAGATCTCTTGAATCTCT |
| IGF2BP3 #1 | GCUGGAGCUUCAAUUAAGATT |
|  | UCUUAAUUGAAGCUCCAGCTT |
| IGF2BP3 #2 | AATTCCTGCAATGGAGATATC |
|  | GATATCTCCATTGCAGGAATT |
| Hur #1 | GGUUUGGGCGGAUCAUCAATT |
|  | UUGAUGAUCCGCCCAAACCTT |
| Hur #2 | GCAGAUGUUUGGGCCGUUUTT |
|  | AAACGGCCCAAACAUCUGCTT |
| HNRNPA2B1 #1 | GAGGTGGTTATGACAACTA |
|  | TAGTTGTCATAACCACCTC |
| HNRNPA2B1 #2 | TGGTCATAATGCAGAAGTA |
|  | TACTTCTGCATTATGACCA |

KIAA1429-siRNA is a gift from Zhang chang[1].

[1] Zhang C, Sun Q, Zhang X, Qin N, Pu Z, Gu Y, Yan C, Zhu M, Dai J, Wang C, Li N, Jin G, Ma H, Hu Z, Zhang E, Tan F, Shen H. Gene amplification-driven RNA methyltransferase KIAA1429 promotes tumorigenesis by regulating BTG2 via m6A-YTHDF2-dependent in lung adenocarcinoma. Cancer Commun (Lond) 2022; 42: 609-626.
